# A sonosensitiser‐based polymeric nanoplatform for chemo‐sonodynamic combination therapy of lung cancer

**DOI:** 10.1186/s12951-021-00804-9

**Published:** 2021-02-25

**Authors:** Yanan Zhang, Abdur Rauf Khan, Xiaoye Yang, Yikang Shi, Xiaogang Zhao, Guangxi Zhai

**Affiliations:** 1 Department of Pharmaceutics, Key Laboratory of Chemical Biology (Ministry of Education), School of Pharmaceutical Sciences, Shandong University, Jinan, 250012 People’s Republic of China; 2 National Glycoengineering Research Center, Shandong University, Jinan, 250012 China; 3 Department of Thoracic Surgery, The Second Hospital of Shandong University, Jinan, 250033 Shandong China

**Keywords:** Chemo-sonodynamic therapy, Rhein, Redox/ultrasound-responsive, Reactive oxygen species, Macrophages

## Abstract

**Background:**

Lung cancer is the most common type of tumour worldwide. Its relative lethality is considerably high. However, since the tumour tissues are located deep within the human body, traditional technologies, such as photodynamic therapy, do not have the desired effect. Sonosensitisers can penetrate deeply into tissues, and sonodynamic therapy (SDT) effectively inhibits tumours by generating reactive oxygen species. Ultrasound can also penetrate deeply, with a favourable tumour inhibition effect.

**Results:**

A redox/ultrasound-responsive Rhein-chondroitin sulphate-based nano-preparation encapsulating docetaxel was fabricated. The nanoparticles displayed increased cellular uptake with quick drug release, good stability, and a monodispersed form in the physiological environment. Rhein induced apoptosis and altered mitochondrial membrane potential, which enhanced the expression of apoptosis-related proteins. SDT inhibited the metastasis and angiogenesis of cancer cells and activated anti-tumour capacity by reducing the expression of M2 macrophages.

**Conclusions:**

The potential of Rhein for SDT was demonstrated. Production of reaction oxygen species was markedly enhanced after ultrasound treatment. The nanoplatform enhanced the synergistic anti-tumour effects of SDT and chemotherapeutic efficacy. The approach was biocompatibility. The findings could inform investigations of chemo-SDT for different cancers. 
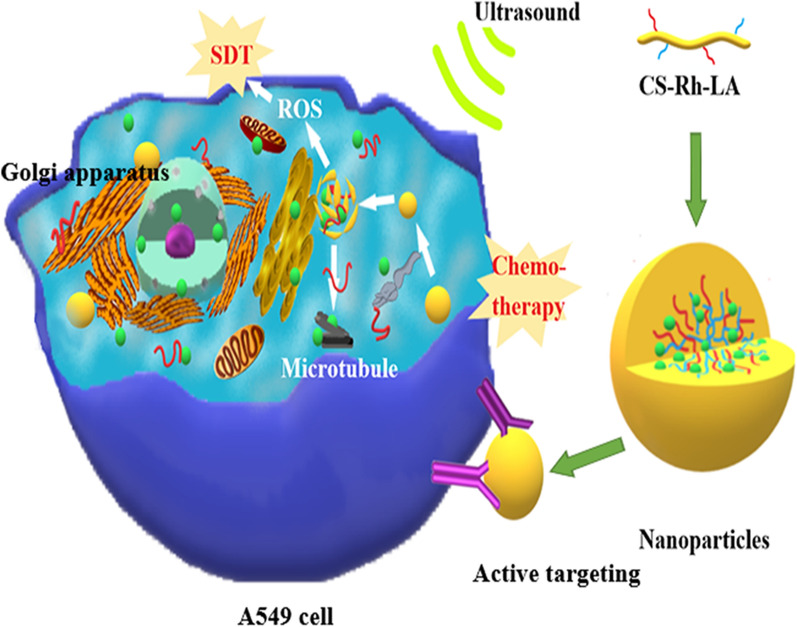

## Introduction

Lung cancer is the most frequently occurring type of tumour-associated death worldwide. Approximately 1.8 million people are diagnosed each year. The lethality rate is 88 % [1]. Non-small cell lung cancer (NSCLC) is the most common form of lung cancer [2, 3]. Among the various therapeutic strategies, combination therapies based on multifunctional nanoplatforms are considered an effective way to eliminate tumours [4, 5]. In addition, many non-invasive technologies, such as near-infrared (NIR) light [6] and ultrasound [7] have been applied to treat cancers. Their safety and efficacy are enhanced compared to traditional chemotherapy and radiation therapy. Compared with the limited penetration depth that was restricted by the laser power, ultrasound energy can penetrate deeper tumour tissues within the bounds of safety. Therefore, the combination of sonodynamic therapy (SDT) and chemotherapy has attracted attention [8].

As previously reported, the principle of SDT is similar to photodynamic therapy. Reactive oxygen species (ROS) are produced under the stimulation of light or ultrasound. This process leads to a singlet-to-triplet transition and apoptosis of tumour cells [9]. ROS initiate oxidisation reactions and ultimately result in irreparable cell damage [10]. To enhance the low efficacy of ROS production by pure ultrasound, the choice of sonosensitisers (SSs) is critical. SSs can increase the level of ROS in a very selective manner [11]. SSs that include protoporphyrin IX [12], hematoporphyrin monomethyl ether (HMME) [13], chlorin e6 (Ce6) [14], titanium dioxide (TiO_2_) [15], and silicon-based [16] nanoparticles have been investigated concerning their influence on ROS production and tumour inhibition [17, 18]. However, the commonly used SSs are expensive and generate ROS only under ultrasonic conditions.

To overcome this drawback, Rhein (Rh) was presently identified as a SS in this study. Rh can directly and gradually generate ROS through the c-Jun N-terminal kinase (JNK)/Jun/caspase-3 signalling pathway, which increases the production of ROS in deeper tissues that are not exposed to ultrasound energy [19]. In addition, Rh favourably inhibited tumours by activating the secretion of apoptosis-related proteins, such as caspase-3, BCL-2 and BAX, in tumour cells after treatment [20]. Rh also inhibits tumour angiogenesis [21, 22]. However, the poor solubility and instability of Rh limit the in vivo therapeutic effect in cancers. Therefore, a stimuli-responsive nanoplatform was constructed to improve the tumour-targeting effect and enhance anti-tumour capability.

We previously demonstrated that chondroitin sulphate (CS), a family member of glycosaminoglycan that targets CD44 receptors, is overexpressed on tumour cells [23–25]. CS also target the Golgi apparatus after the cellular uptake process [26]. CS was selected as the backbone of the developed nanoplatform. Rh was grafted onto the CS backbone with adipic dihydrazide (ADH) molecules to aminate CS. The stable amphiphilic polymer CS-ADH-Rh was formulated. In addition, lipoic acid (LA) as a crosslinking agent, was also grafted to the backbone to form intermolecular disulfides when exposed to a catalytic amount of dithiothreitol (DTT) in nitrogen. During the process, intramolecular disulfide bonds broke, and intermolecular disulfides formed [27]. The nanoparticles were cross-linked and endowed the nanoplatform with redox/ultrasound-sensitive activity and stability. The amphipathic self-assembled nanoplatform could rapidly release the encapsulated drug in the highly reductive environment of the cytoplasm [28–31]. In addition, the hydrophobic anti-tumour drug docetaxel (DTX), which has poor solubility and is very toxic, was easily loaded into the hydrophobic segment of the nanoplatform. The incorporation of DTX could play a role in combined therapy by inducing G2/M phase arrest in mitotic cell division and destroying the microtubule structure of tumour cells [32, 33].

**Scheme 1:**
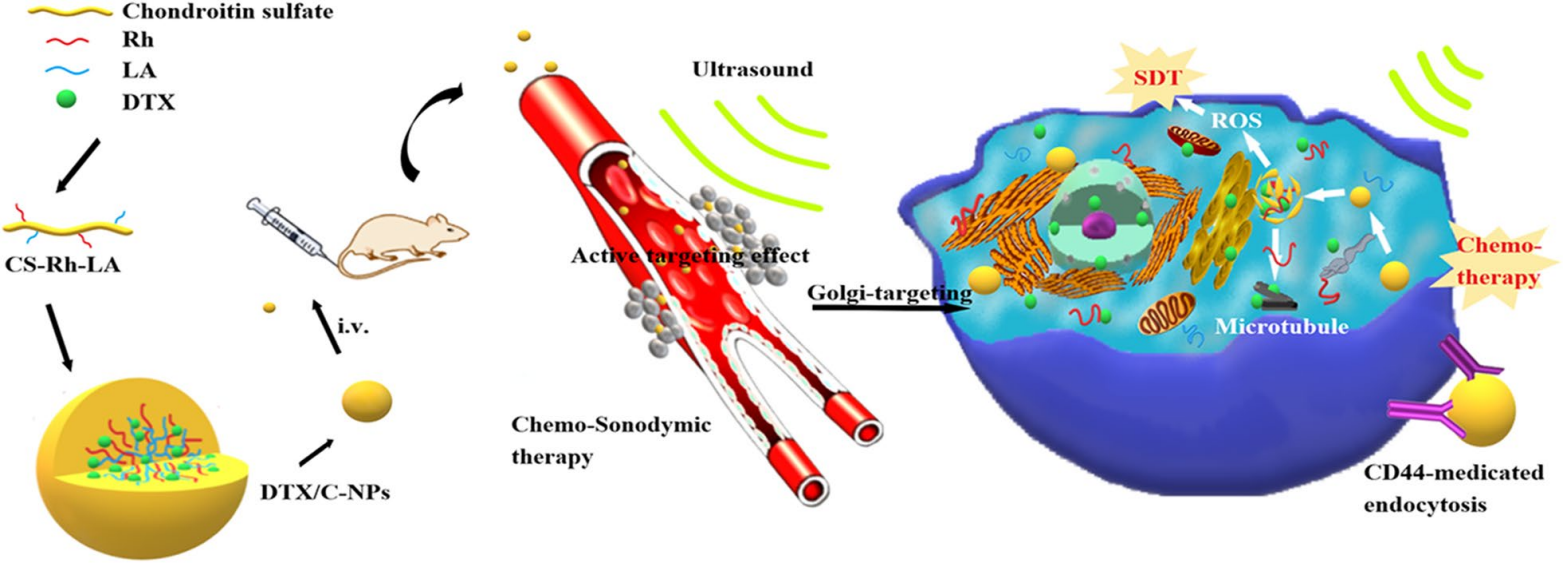
**a** Synthesis and self-assembly of DTX/C-NPs. **b** The accumulation and ultrasound-triggered ROS production in tumour cells.  DTX/C-NPs induce cell cycle arrest in A549 cells by facilitating microtubule polymerisation. **c** Illustration of the combined chemo-SDT therapy of the redox /ultrasound-responsive nanoparticles

Consequently, we synthesised a redox/ultrasound-sensitive DTX-loaded CS-ADH-Rh-LA nanoplatform to inhibit tumour growth and metastasis through combined chemo-SDT. Rh in the nanoparticles induced a favourable SDT effect both in vivo and in vitro, and significantly suppressed tumour invasion and migration. In addition, the nanoparticles could target the Golgi apparatus and facilitate structural damage of organelles, especially during SDT. In addition, the nanoparticles successfully enhanced ROS generation in time-and concentration-dependent manners after entry into A549 cells, and could effectively inhibit the invasion and migration of cancer cells. The nanoplatform improved the induction of apoptosis in tumour cells and activated the immune system after SDT. Therefore, the role of Rh as an SS was discovered and exploited in this highly efficient and economic nanoplatform. The findings highlight a new approach for the development of novel SSs (Scheme 1).

## Result and discussion

### Synthesis and characterization of nanoparticles

**Fig. 1:**
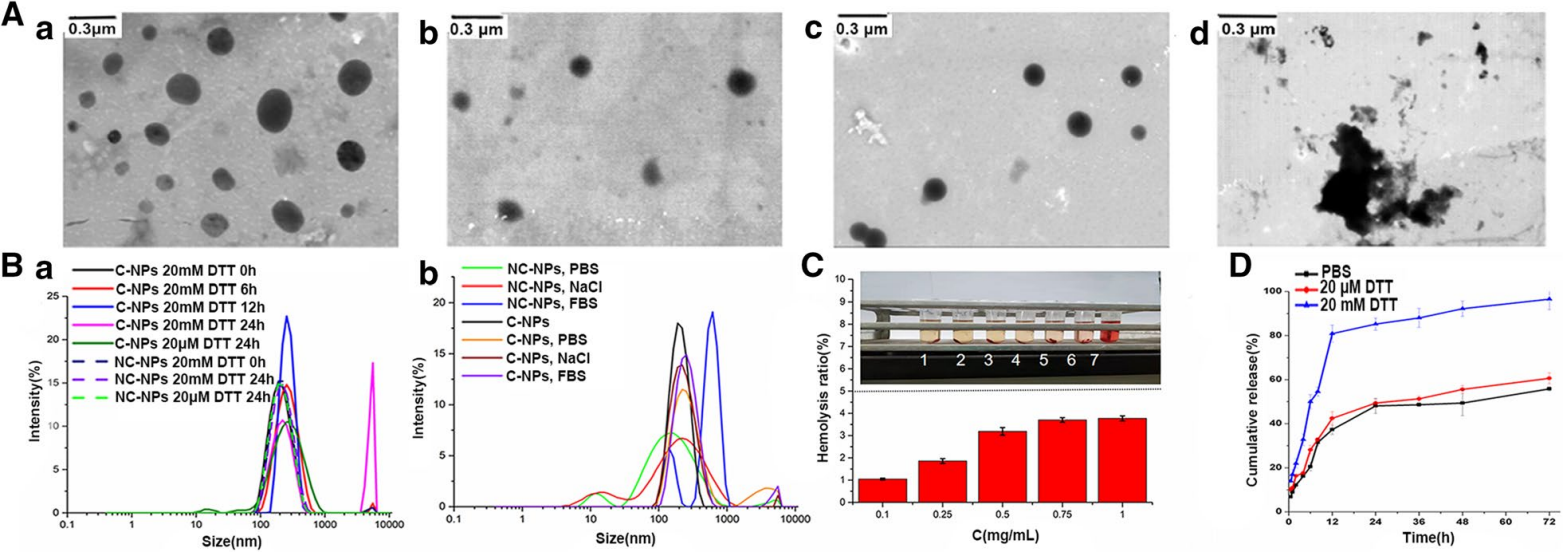
**A** Transmission electron microscopy of the morphology of NC-NPs (a) and C-NPs (b) dispersed in distilled water . The particle size distribution of C-NPs after 24 h of incubation with 20 µM dithiothreitol (DTT) (c) and 20 mM DTT (d). **B **(a) The responsive disassembly of C-NPs and NC-NPs. Particle size was determined at different times in the reductive environment in the presence of DTT. (b) Crosslinking stability of nanoparticles. Size changes of C-NPs and NC-NPs in various solutions of phosphate buffered saline, NaCl and 10% foetal bovine serum. **C** Haemolysis rate of C-NPs (number 1–7 represents the groups of blank control, 0.1, 0.25, 0.5, 0.75, 1 mg/mL C-NPs and positive control). **D** Cumulative DTX release from the C-NPs. *p < 0.05, **p < 0.01

Nanoparticles of CS-ADH-Rh-LA (NC-NPs and C-NPs representing non-crosslinking and crosslinking nanoparticles, respectively) were prepared by a simple sonication method. The size ranged from 158.4 to 192.4 nm (Table 1). Although Rh was hydrophobic part, NC-NPs and C-NPs increased in size with increased Rh to CS-ADH ratio, probably owing to the steric hindrance effect of Rh molecules. The size of CS-ADH-Rh was 215.2 nm, which was significantly larger than the size of NC-NPs, indicating the enhanced self-assembled effect after LA was grafted. The results showed that the critical aggregation concentration value of NC-NPs with different ratios of Rh to CS-ADH ranged from 33.74 to 66.54 µg/mL, which indicated excellent self-aggregation ability and dilution stability of NC-NPs (Table 1). Compared with NC-NPs, C-NPs were smaller with better dispersion behaviour (Fig. 1A(a) and (b)). The findings validated our hypothesis that the crosslinking process could improve the stability of the nanoparticles [34]. Both NC-NPs and C-NPs exhibited good stability with a zeta potential of -21.0 mV and −28.9mV, respectively (Table 1). ^1^H-numclear magnetic resonance (NMR), ^13^ C-NMR, and Fourier transform infrared (FT-IR) results of CS-ADH-Rh-LA are shown in Additional file 1: Fig. S2.

**Table 1:** Characteristics of CS-Rh-LA nanoparticles

		NC-NPs				C-NPs			
Sample	Rh DS (%)	Diameter (nm)	PDI	ζ (mV)	CMC (µg/mL)	Diameter (nm)		PDI	ζ (mV)
CS-Rh-LA1	0.93	165.7 ± 4.23	0.153 ± 0.015	−15.6	66.54	138.5 ± 3.50		0.128 ± 0.018	−25.7
CS-Rh-LA2	2.69	180.6 ± 3.90	0.166 ± 0.029	−18.8	47.47	163.8 ± 3.09		0.122 ± 0.022	−26.8
CS-Rh-LA3	3.15	184.4 ± 2.50	0.100 ± 0.014	−21.0	33.74	178.3 ± 2.65		0.0913 ± 0.008	−28.9

Values are listed as Mean ± SD, n = 3

### Stability of nanoparticles

To evaluate the redox-responsive properties of C-NPs, the reductive stability was also evaluated in different concentrations of dithiothreitol (DTT) (Fig. 1B(a)). C-NPs increased in size from 180.8 nm to 272.5 nm, 401.6 nm and 715.0 nm after 6, 12 and 24 h of incubation with 20 mM DTT, respectively. Transmission electron microscopy (TEM) results (Fig. 1A(c) and (d)) revealed the gradual destruction of C-NPs. Crushed NPs were evident after 24 h of incubation in 20 µM DTT. However, there was negligible change in the size of C-NPs incubated with 20 µM DTT solution (Additional file 1: Fig. S3). In addition, there was little change in the size of NC-NPs after incubation with DTT solutions. Further, in vitro stability was evaluated after incubation with phosphate buffered saline (PBS; pH 7.4, 10 mM), NaCl, and complete medium with 10% foetal bovine serum (FBS) for 24 h (Fig. 1B(b)). In both PBS and NaCl solutions, NC-NPs decreased in size, while C-NPs maintained favourable colloidal stability. In particular, the stability of C-NPs was better than that of NC-NPs in 10% FBS solution for 6 days (Additional file 1: Fig. S4). In addition, to estimate blood stability, the haemolysis rate of C-NPs was studied (Fig. 1C). The haemolysis rate increased with the concentration of C-NPs, but the rate was always below 5% (0.1 to 1 mg/mL), indicating the good biocompatibility of the synthesised polymers. Therefore, C-NPs possessed superior colloidal stability after crosslinking in normal physiological environments or blood, and disassembled quickly in a simulated tumour microenvironment, guaranteeing their successful delivery.

### Drug loading and release assay

To explore the combined therapy, docetaxel (DTX) was encapsulated in C-NPs by the sonication-dialysis method, and the effect of DTX on drug loading was studied, as shown in Table 2. With an increase in the charge ratio of DTX to C-NPs of 1:10 to 5:10, C-NPs could carry more DTX. Thus, the drug loading (DL) increased from 2.50 to 12.05%. The corresponding encapsulation efficiency (EE) increased from 23.93 to 35.73% as the ratio increased from 1:10 to 3:10, but decreased to 28.25% at a 5:10 ratio. This could be explained by the breakage of the hydrophobic-hydrophilic balance for the superfluous addition of DTX. In addition, the size increased as the proportion of DTX. However, the zeta potential had the highest absolute value only at a ratio of 3:10. The DTX to C-NPs ratio of 3:10 was selected for further studies. After crosslinking, the in vitro release profile of DTX/C-NPs was further evaluated in PBS alone and PBS containing 20 µM or 20 mM DTT (Fig. 1D). The cumulative release of DTX in PBS containing 20 mM DTT was more rapid in the first 12 h (nearly 80%) than in PBS containing 0 and 20 µM DTT. The release reached approximately 93% compared to 56% and 53% for 20 µM DTT or PBS groups, respectively, at 72 h. Therefore, the drug delivery system could self-dissociate quickly and release the drug as the environment changed.

**Table 2:** Characteristics of DTX/CS-ADH-Rh-LA nanoparticles

Sample	DTX / polymer	DL (%)	EE (%)	Diameter (nm)	ζ (mV)
CS-ADH-Rh-LA	1 : 10	2.50 ± 0.18	23.93 ± 2.26	193.5 ± 1.2	−24.2
	2 : 10	5.71 ± 0.15	31.75 ± 2.11	201.7 ± 2.2	−25.2
	3 : 10	9.57 ± 0.38	35.73 ± 1.97	231.7 ± 3.9	−30.7
	5 : 10	12.05 ± 0.25	28.25 ± 1.37	254.0 ± 2.3	−28.9

All values are presented as Mean ± SD (n = 3)

### Cellular uptake process

**Fig. 2:**
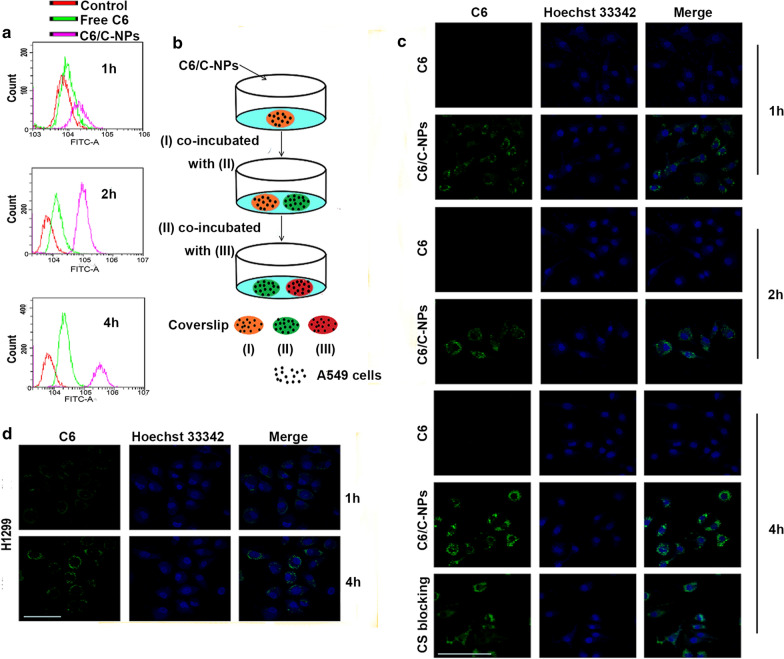
Cellular uptake efficiency and intracellular delivery. **a** Flow cytometry analysis of free C6, C-NPs and C-NPs after CS blocking of A549 cells for various times. **b** Mechanism of intracellular uptake of C-NPs observed by CLSM. A549 cells on a coverslip (I) were incubated in C6/C-NPs medium for 4 h. The coverslip (I) was washed, photographed and placed together with another coverslip (II) in a dish filled with fresh medium for 12 h. The process was repeated on another coverslip (III). All results of the three coverslips are presented in Additional file 1: Fig. S4. Scale bars denote 100 µm. **c** Confocal microscopy of cellular uptake of A549 cells incubating with different solutions. Scale bar denotes 100 µm. **d** CSLM images of H1299 cells incubated with C-NPs for 1 and 4 h. Scale bar denotes 100 µm

Flow cytometry (FCM) and confocal laser scanning microscopy (CLSM) were both used to analyse the internalisation of coumarin 6 (C6)-loaded C-NPs prepared by the same method as DTX/C-NPs. The fluorescent-labelled cells were screened out, followed by compilation of the statistical data as a histogram (Fig. 2a). C-NPs internalised in A549 cells in a time-dependent manner. The fluorescent signal became the strongest after 4 h of incubation. The internalisation behaviour of the free C6 group appeared similar. However, the fluorescence intensity was much weaker than that of the C-NPs group. The cellular uptake mechanism was studied using CLSM, as shown in Fig. 2c. C6-loaded C-NPs were compared with free C6 and CS blocking groups (A549 cells were treated with CS solutions in advance) at different time points. Similar to the FCM result, the fluorescence of C6/C-NPs was stronger than that of the C6 free group, while no signal was detected in the control group. The internalising capacity of the CS blocking group was between that of the control and free C6 groups, indicating the successful blocking of CD44 receptors and confirming the active targeting-mediated internalisation of C-NPs. For H1299 cells, the fluorescence intensity was much weaker than that of A549 cells (Fig. 2d), confirming the higher cellular uptake of A549 cells, which have numerous CD44 receptors.

The exocytosis-dependent transcellular transport of C6-loaded C-NPs was measured in batches of stimulated A549 cells. The fluorescence intensity of C6 was high and readily evident in cells on coverslips (I) and (II) (Additional file 1: Fig. S5). The findings indicated that C6 taken up in the first step was secreted into the fresh medium and then absorbed by cells on coverslip (II). However, a fluorescent signal was barely evident in coverslip (III). This might be attributed to the reduction of the fluorescent material. Collectively, C-NPs were actively taken up by cancer cells due to the overexpression of the CD44 receptor, and the intake increased with time. In addition, the C-NPs could enter the cells via endocytosis and were delivered to the surrounding cells through exocytosis, which might lay the foundation for active penetration.

### Cytotoxicity and apoptosis

**Fig. 3:**
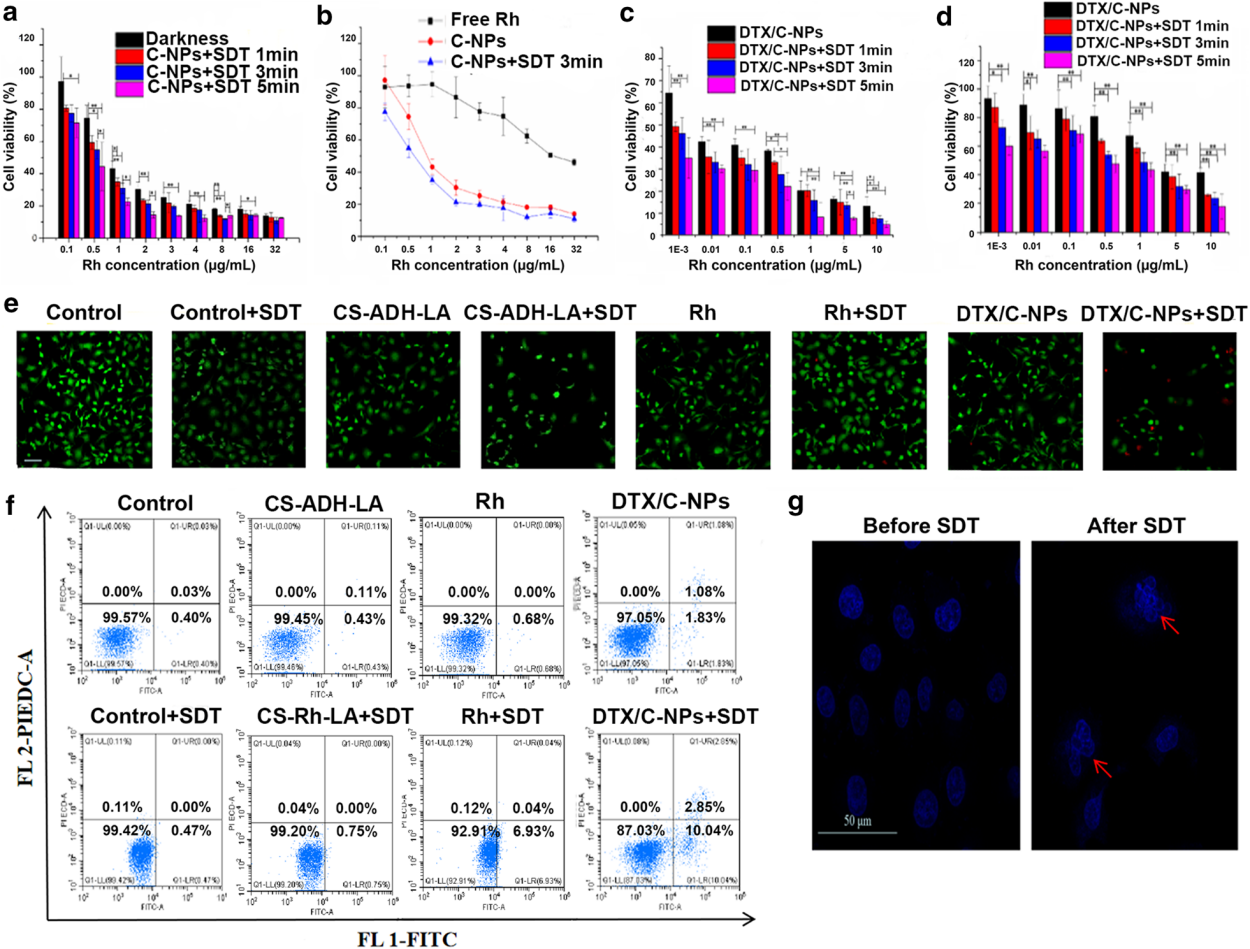
Assessments of in vitro cytotoxicity and apoptosis. **a** Viability of A549 cells treated with different concentrations of C-NPs to A549 cells in the dark with ultrasonication at 1.2 W/cm^2^ for various times. **b** Viability following treatment of A549 cells with different concentrations of Rh, C-NPs and C-NPs + SDT followed by ultrasonication at 1.2 W/cm^2^ for 3 min. **c** and **d** Viability of A549 cells (**c**) and H1299 cells (**d**) following treatment with DTX/C-NPs along or with SDT using ultrasonication at 1.2 W/cm^2^ for different times. **e** Fluorescence images of live/dead cells using Calcein-AM and PI co-staining of A549. Scale bar denotes 50 µm. **f** FCM examination of cell apoptosis using the different treatments. **g** Morphologies of cell nucleus before or after SDT therapy. *p < 0.05, **p < 0.01. Scale bar denotes 50 µm

The A549 cell cytotoxicity and apoptosis induced by C-NPs and DTX/C-NPs were evaluated. First, the CCK-8 method was used to estimate the cytotoxicity of C-NPs. Cells were incubated with C-NPs in different concentrations of Rh. The cells were then treated using ultrasound (1.2 W/cm^2^) for 0, 1, 3, and 5 min (darkness, SDT-1, SDT-3, and SDT-5, respectively). C-NPs were negligibly cytotoxic in the dark environment at 0.1 µg/mL (Fig. 3a). The cytotoxicity in the darkness condition increased with increasing Rh concentration and showed a similar trend to that of SDT groups at 32 µg/mL. However, the SDT groups exhibited significantly enhanced cytotoxicity (p < 0.01) with increasing length of ultrasonication. The IC50 values for the dark, SDT-1, SDT-3, and SDT-5 groups were 1.061, 0.5664, 0.5049, and 0.2959 µg/mL, respectively. As shown in Fig. 3b, C-NPs and DTX/C-NPs showed remarkable cell killing ability at low concentrations compared with free Rh under the same environment. This could likely be attributed to the active targeting of the C-NPs. DTX/C-NPs manifested a stronger cell killing effect than the C-NPs after SDT treatment, especially at low concentrations.

The synergistic cytotoxicity of DTX/C-NPs against A549 and H1299 cells was studied (Fig. 3c and d). For A549 cells, DTX/C-NPs showed an excellent cell killing effect, even at low concentration of 10^− 3^ µg/mL in darkness, with cell viability of 64.3% compared with the 93.8% viability of H1299 cells. The results indicated the preferably active targeting of CS to CD44 receptors expressed on A549 cells, rather than H1299 cells, and also the reduced cytotoxicity of DTX/C-NPs compared to free DTX solution (Additional file 1: Fig. S6). The cytotoxicity of C-NPs nearly doubled after encapsulation of DTX with or without SDT treatment in A549 cells. The cell viability of the CS-ADH-LA polymer exceeded 80% at all concentrations, indicating the safety of the materials (Additional file 1: Fig. S7). Considering the collective results of the effective cytotoxicity and shorter ultrasonic time, A549 cells treated with SDT for 3 min were used for further experiments. The efficacy of DTX/C-NPs along with SDT was evaluated using a live/dead cell staining study with calcein-AM at excitation and emission wavelengths of 490 and 515 nm, respectively, together with propidium iodide (PI; excitation and emission wavelengths of 517 and 617 nm, respectively) (Fig. 3e). Red fluorescence emitted by dead cells emerged and became stronger after incubation with DTX/C-NPs after SDT treatment compared with the control group, demonstrating the favourable ROS-generating property. However, no red fluorescence was observed for the free Rh group compared to the Rh + SDT group, indicating the efficiency of SDT following Rh treatment.

FCM revealed that ultrasound or Rh alone induced negligible apoptosis and necrosis in A549 cells (mortality ratio < 1%). The apoptosis rates of the SDT control group and CS-ADH-LA were similar (Fig. 3f). Mortality of free Rh with SDT (6.9% cell apoptosis rate) was enhanced compared to the control group, but was lower than that of the DTX/C-NPs + SDT group (10.0% apoptosis rate and 2.9% rate of necrosis). The more pronounced mortality of Rh in the SDT group than the DTX/C-NPs group highlighted the importance of Rh and SDT treatment in tumour suppression in A549 cells. SDT induced distinct karyopyknosis and karyolysis compared with the group without SDT treatment after culturing with DTX/C-NPs for the same time (Fig. 3g).

### ROS detection during SDT

**Fig. 4:**
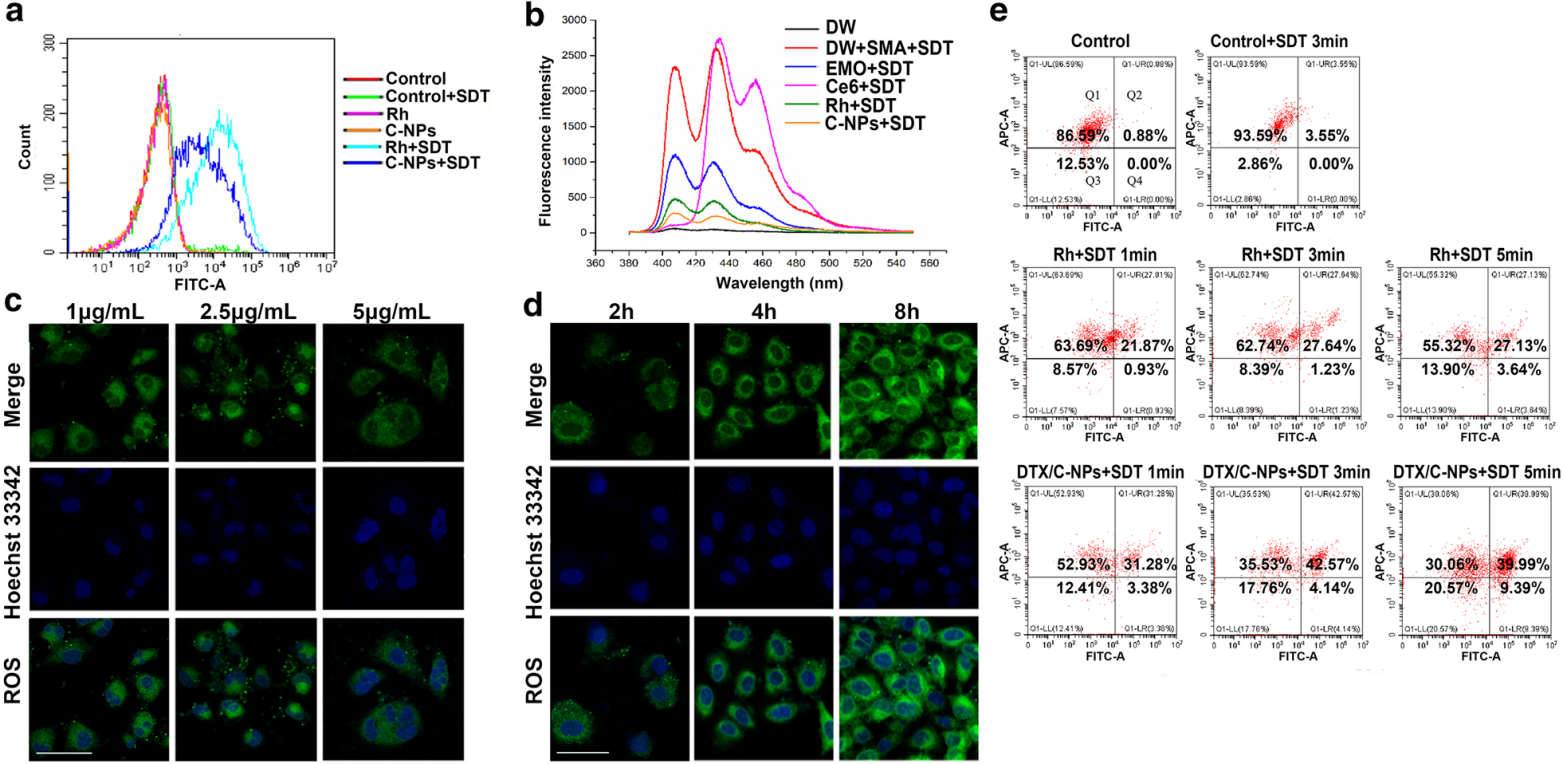
ROS production and mitochondrial membrane potential (MMP) assessment. **a** FCM results of ROS generation assay in different solutions with/without SDT. **b** The production of singlet oxygen was detected in various components by DMA after SDT treatment. **c** and **d** ROS production was observed using 2’-7’dichlorofluorescin diacetate as a probe by CLSM under (**c**) different concentrations of C-NPs (containing 1, 2.5 or 5 µg/mL Rh) at 12 h or (**d**) at different incubation times with fixed concentration of C-NPs (5 µg/mL Rh). Scale bar denotes 50 µm. **e** MMP measurement result using JC-1 cationic carbocyanine dye as the probe. The Y-axis represents the cells with normal MMP (red fluorescence) transferred towards the X-axis when the MMP decreased (green fluorescence)

The generation of ROS is considered crucial for apoptosis and cellular component destruction of cancer cells [35]. Presently, this was mainly attributed to the addition of Rh and SDT. The formation of intracellular ROS was detected in A549 cells using CLSM and FCM after different incubation times. After 4 h, the Rh + SDT group manifested obvious green fluorescence compared with the Rh group. The fluorescence of N-acetyl-L-cysteine (NAC, the ROS scavenger) and the control group was negligible (Additional file 1: Fig. S8). However, the Rh + SDT and C-NPs + SDT groups displayed similar ROS-producing capacities (Additional file 1: Fig. S8 and Fig. 4a). The finding was probably due to the short incubation time that limited the release of ROS because the NPs were not degraded sufficiently. To prove the correlation between effective SDT and free Rh, the singlet oxygen contents were detected with different agents using 9, 10-dimethylanthracene (DMA) after sonication for 4 h. The Rh and C-NPs peaks decreased significantly compared with Ce6, emodin (EMO) and the control group. Free Rh generated more singlet oxygen than C-NPs (Fig. 4b). The findings indicated that the crosslinking of C-NPs limited the speed of release of the ROS generated by Rh molecules. This phenomenon may have been relieved in tumour cells because of the hydrolysis of disulfide bonds. To verify this idea, fluorescence images of ROS with different concentrations of C-NPs or various incubation times were observed (Fig. 4c and  d). As expected, ROS were produced in time-and concentration-dependent manners. Fluorescence was less in the presence of 5 µg/mL C-NPs after 12 h of incubation. This might be attributed to the apoptosis of A549 cells at the high concentrations.

### Detection of mitochondrial membrane potential (MMP)

The change in MMP indicates the destruction of the adenosine triphosphate (ATP)-production process by utilising the proton electrochemical gradient potential across the mitochondrial membrane [36]. We used the JC-1 assay kit to determine the variation of MMP by FCM. The Y- and X-axis represent MMP in normal cells (red fluorescence) and abnormal cells (green fluorescence), respectively. The graph is divided into four parts, in which Q1 and Q4 represent pure fluorescence positive for red or green, and Q2 or Q3 as double positive or double negative, respectively. Control groups with/without SDT were predominantly located in the Q1 zone, indicating the formulation of JC-1 aggregates with high MMP levels in cells. However, the MMP was decreased in the Rh + SDT and C-NPs + SDT groups, indicating the rupture of the mitochondrial membrane and the disruption of JC-1 aggregates. The destruction of the mitochondrial membrane was increased with increased SDT time (Fig. 4e). The green fluorescence ratio (sum of Q2 and Q4 zones) was 0.9% and 3.6% for the control and control + SDT groups, respectively, while the ratio increased to 30.6% and 49.4% for the Rh and DTX/C-NPs groups after sonication for 5 min. The findings indicated that ultrasound can damage the mitochondrial membrane, leading to cellular apoptosis in a time-dependent manner with the help of SSs.

### DTX/C-NPs induce cell cycle arrest in A549 cells by facilitating microtubule polymerisation

**Fig. 5:**
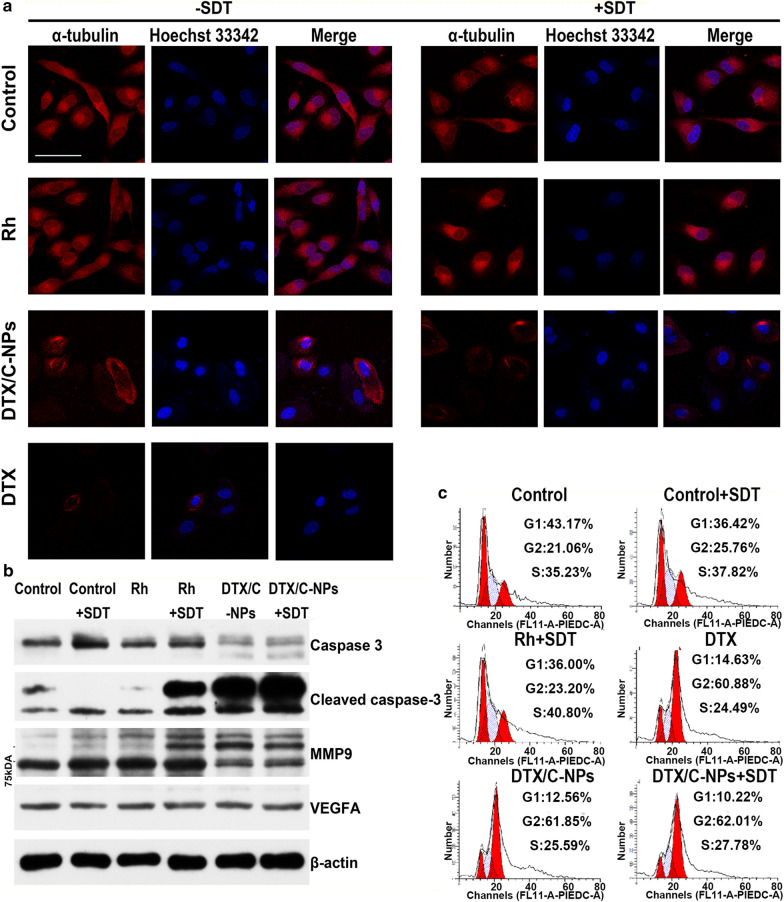
**a** A549 cells incubated with different solutions with/without SDT treatment were fixed and stained with α-tubulin (red) antibodies. Fluorescence images were acquired by CLSM. Scale bar denotes 50 µm. **b** Western blot results. Expressions of caspase-3, cleaved caspase-3, MMP9 and VEGFA proteins after SDT treatment. **c** Cell cycle detection of A549 cells was measured using a kit after incubating with different solutions with/without SDT treatment

To study the growth inhibition effect of DTX/C-NPs on A549 cells, the microtubule morphology and cell cycle were analysed. Microtubules marked by α-tubulin protein displayed a clear morphology and were evident throughout A549 cells. However, the microtubules disappeared and formed polymers in certain regions of A549 cells after culture with DTX or DTX/C-NPs. The findings indicated the cytotoxicity and inhibition of cell division by DTX. SDT facilitated microtubule polymerisation to a certain extent. Polymerisation was weak, as the images of control and Rh + SDT showed brighter red fluorescence in some parts of the cell (Fig. 5a). The influence of DTX/C-NPs on cell cycle arrest was further investigated using a DNA detection kit with PI staining (Fig. 5c). Compared to the control group, the percentage of G2/M cells increased from 21.06 to 23.20%, 25.76 %, 60.88 %, and 62.01 % for the Rh + SDT, control + SDT, DTX and DTX/C-NPs + SDT groups, respectively. Therefore, A549 cells could be arrested in the G2/M phase by promoting microtubule polymerisation or preventing microtubule depolymerisation with DTX/C-NPs and SDT.

### Western blot analysis

Apoptosis-related proteins were measured by the western blot assay (Fig. 5b). Since apoptosis is related to poly ADP-ribose polymerase cleavage, the caspase-3 and activated caspase-3 hallmark proteins were detected. Compared with the control group, the DTX/C-NPs + SDT group expressed the minimum amount of caspase-3 protein, but the maximum amount of cleaved caspase-3 protein. Additionally, the expression of matrix metalloproteinase 9 (MMP9) was determined. MMP9 is an enzyme with an affinity to zinc. The enzyme catalyses the degradation of the extracellular matrix and promote the invasion of cancer cells [37]. The expression of MMP9 decreased after incubation with DTX/C-NPs with/without SDT compared to the control and Rh-treated groups. The findings were evidence of the good anti-metastatic activity of DTX/C-NPs and SDT. However, there was little change in vascular endothelial growth factor A (VEGFA) protein expression among different groups. This finding indicated that DTX/C-NPs could not inhibit tumour angiogenesis significantly, probably because of the low Rh concentrations during the therapy.

### Subcellular localisation and distribution

**Fig. 6:**
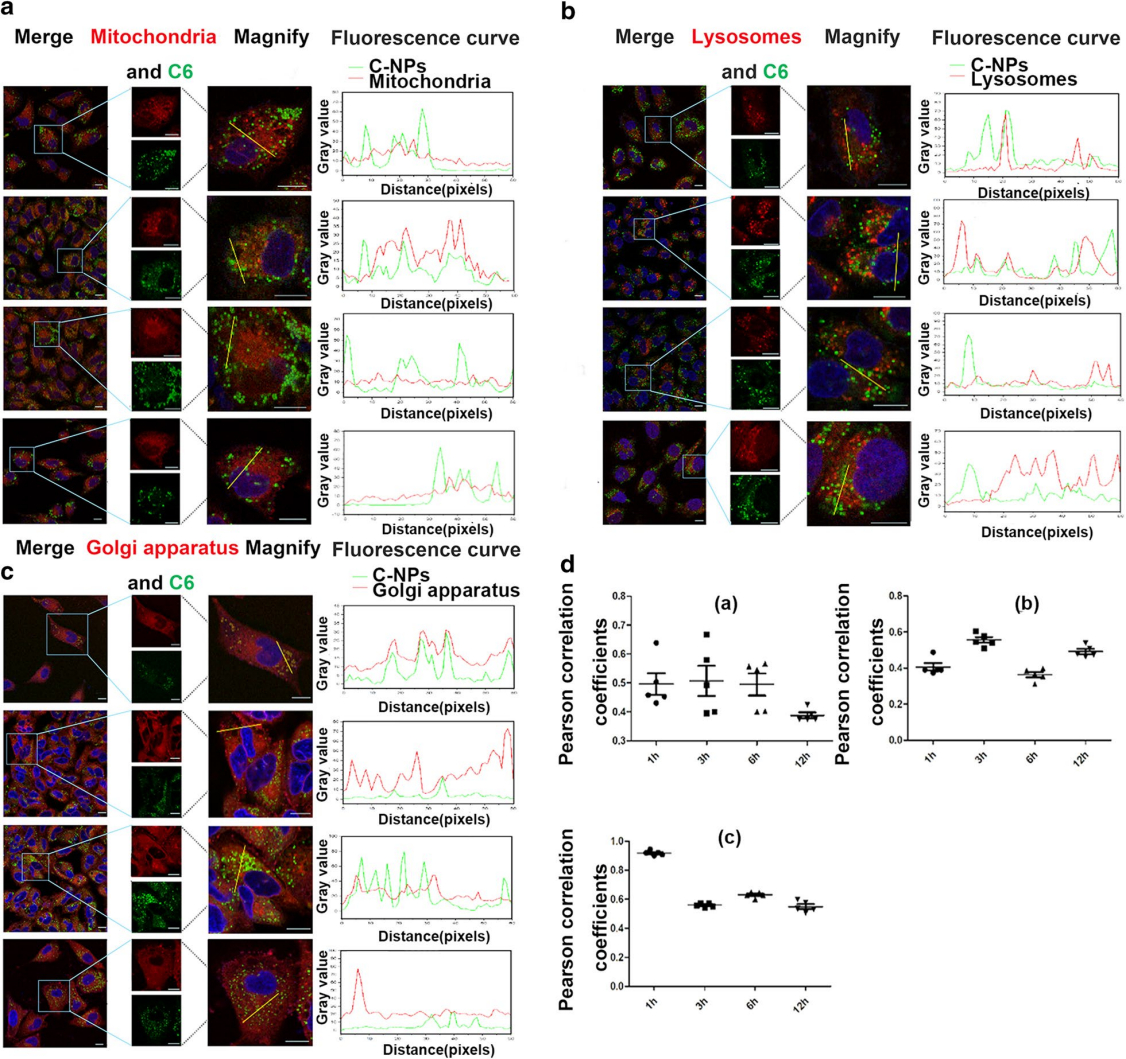
Distribution of C-NPs in several cytoplasmic organelles of A549 cells. The distribution of C6 from C-NPs in mitochondria (**a**), lysosomes (**b**) and Golgi apparatus (**c**) of A549 cells observed by magnified images acquired by CLSM. Fluorescence curves are depicted along the yellow thread. Scale bar denotes 10 µm. **d** The Pearson correlation coefficient of C6 distribution in mitochondria, lysosomes and Golgi apparatus was calculated by Image J software

Subcellular localisation and distribution were studied using C6/C-NPs. After staining with Lyso-Track Red, MitoTracker Red CMXRos and Golgi-Tracker Red to visualise lysosomes, mitochondria and Golgi apparatus, respectively, cells were observed at different time points (Fig. 6). Few C6 signals were co-localised with lysosomes and mitochondria. Their maximum Pearson correlation coefficients were 0.56 and 0.51 after a 3-h incubation, respectively (Fig. 6A, B, Da and b). In contrast, C-NPs rapidly co-localised with the Golgi apparatus after the NPs were internalised into the cells for 1 h. The Pearson correlation coefficient reached 0.92 (Fig. 6C and Dc). The findings provided preliminary evidence that the internalised C-NPs were transported to the Golgi apparatus within 1 h, and were gradually transferred to lysosomes for further enzymolysis between 1 h and 3 h after co-localisation to the Golgi membrane reached saturation. However, for mitochondria, the Pearson correlation coefficient showed little difference at 1, 3, and 6 h, but was significantly decreased at 12 h, which might be attributed to the rupture of the mitochondrial membrane after culturing for 12 h.

### Destruction of golgi structure

**Fig. 7:**
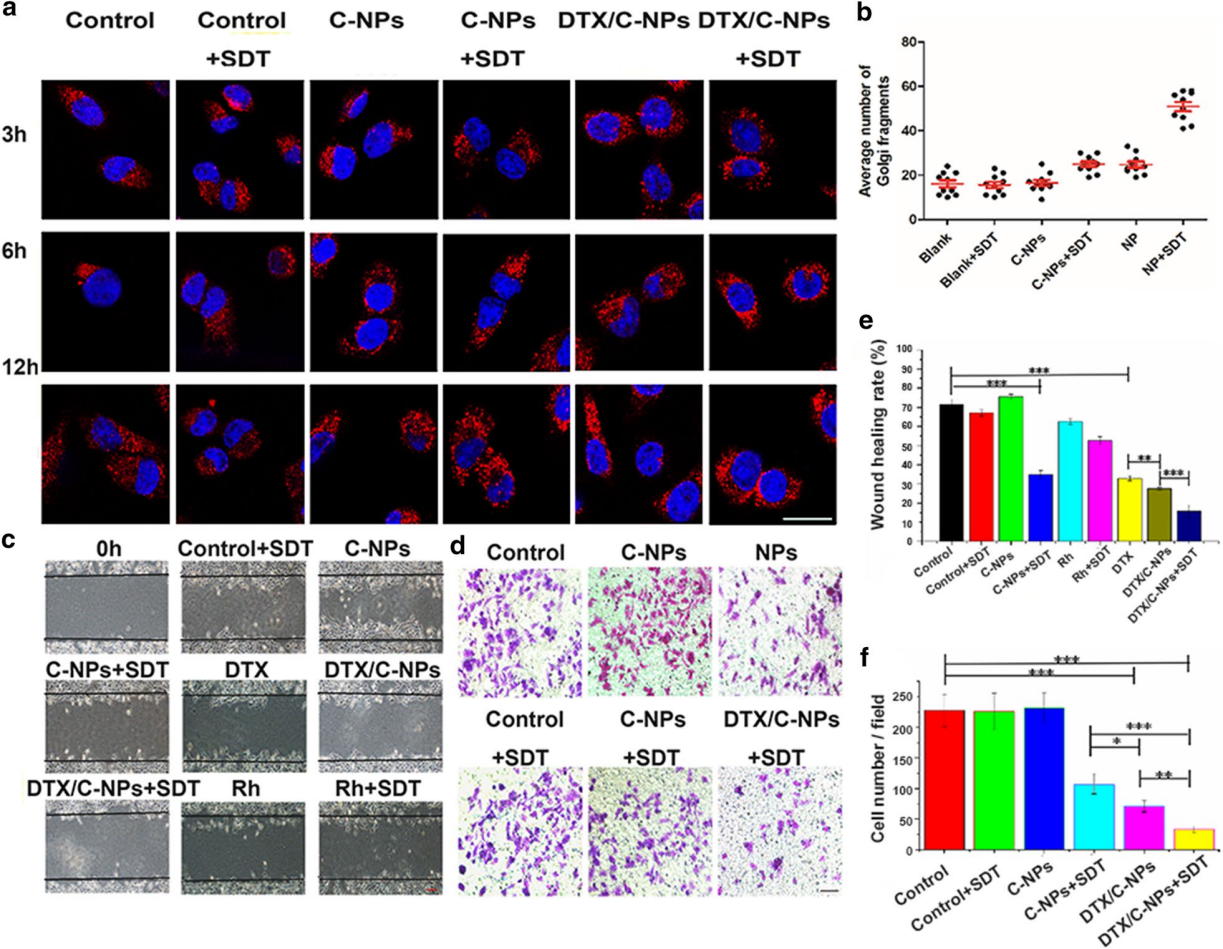
**a** Integrity of Golgi apparatus structure incubated with different preparations with/without SDT treatment. GM130 immunofluorescence (red) was observed by CLSM at different times. Scale bar denotes 50 µm. **b** Fragmented quantitation in Golgi apparatus for A549 cells after different treatments. Each dot represents the number of Golgi bodies from a single cell. **c** Wound healing ability of A549 cells incubated in the absence and presence of SDT for 24 h. Samples were observed by inverted fluorescence microscopy. Scale bar denotes 50 µm. **d** A549 cells that migrated across membranes in Transwell chambers were photographed by inverted fluorescence microscopy. Scale bar denotes 50 µm. The results of wound healing assay (**e**) and Transwell assay (**f**) were both quantified by Origin 9 software

The Golgi apparatus is a hub for sorting and trafficking of intracellular proteins and supports cellular trafficking by balancing proteins and membranes [38]. Presently, the destruction of Golgi structures harmful to tumour growth was observed by immunofluorescent staining. The stability of the Golgi tethering protein GM130 (Golgi matrix protein) was investigated. GM130 in the DTX/C-NPs + SDT group showed obvious depolymerisation as the number of red small spots increased (Fig. 7a). The small spots were counted, and the average number was calculated after incubation for 12 h (Fig. 7b). The number of GM130 proteins was in the order of Rh ≈ Control (±SDT) < DTX/C-NPs ≈ Rh (+ SDT) < DTX/C-NPs (+ SDT). The findings indicated the increasing disruption of Golgi structures among the groups that would lead to increased migration, invasion and poor prognosis of lung cancer [39].

### Inhibition of cell migration and invasion

Migration and invasion are crucial for the evaluation of the metastatic cascade in cancer cells. Presently, a wound healing and Transwell invasion assays were performed to evaluate the migration and invasion, respectively, of A549 cells treated with different solutions at their IC50 concentrations. The scratch test revealed that the wound closure of the control group reached 71.5% after 24 h, but was only 67.1% after SDT treatment. The rate of wound closure was slightly increased (75.5%) in the C-NP solution, but was significantly reduced (34.9%) after SDT treatment. The findings indicated the inefficient inhibition of A549 migration by C-NPs. The wound closure values of the Rh, Rh + SDT, DTX, DTX/C-NPs and DTX/C-NPs + SDT treatment groups were decreased (62.6%, 52.7%, 32.9%, 27.5% and 15.8%, respectively). The results indicated favourable effects of C-NPs and DTX/C-NPs on lessening metastasis, especially when combined with SDT (Fig. 7c and d). The Transwell invasion assay results showed a similar trend (Fig. 7e and f). The average number of cells that crossed the Matrigel was 227.4 ± 25.9 and 225.6 ± 25.6 for control and control + SDT groups, respectively. The C-NPs group showed similar invasion ability with the control group (231.4 ± 23.8), but the number of migrating cells was considerably decreased after SDT (106.2 ± 17.4). The number of migrating cells in the DTX/C-NPs group declined in the absence of SDT (71.2 ± 9.4) and presence of SDT (33 ± 5.4). The findings indicated the important roles of SDT and DTX/C-NPs in inhibiting cell migration and invasion.

### Biodistribution of DiR/C-NPs

**Fig. 8:**
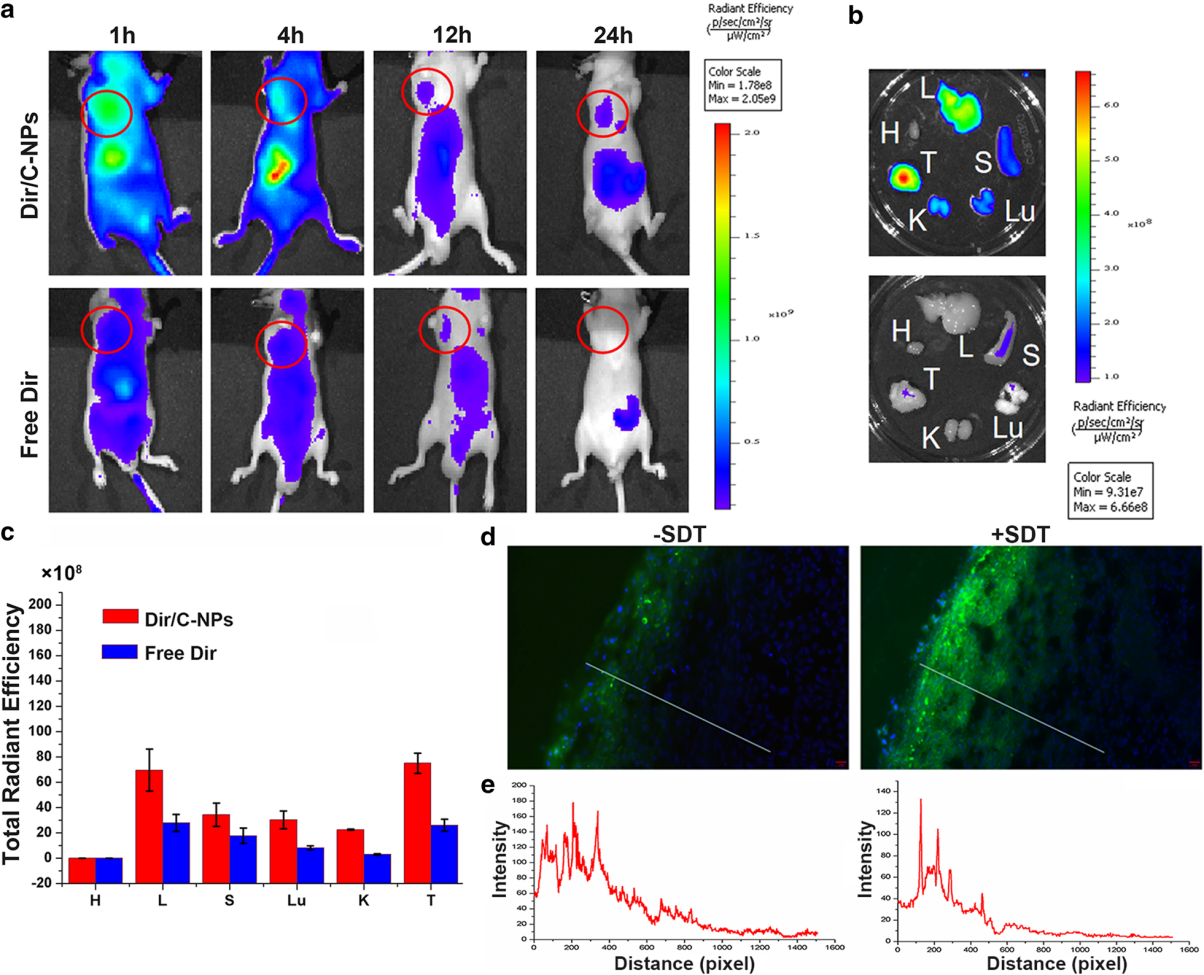
**a** In vivo biodistribution of A549 tumour-bearing nude mice intravenously injected with DiR/C-NPs or free DiR solution 1, 4, 12 and 24 h after injection. The tumour foci are marked with red circles. **b** Ex vivo biodistribution 24 h after intravenous injection of DiR/C-NPs or free DiR for tumour (T) and major organs of heart, liver, spleen lung and kidney (abbreviated respectively as H, L, S, Lu and K). **c** General resident efficiency of organs removed 24 h following the injection of DiR/C-NPs or DiR. **p < 0.01. **d** Fluorescence distribution of C6/C-NPs in A549 solid tumour with/without ultrasound treatment. Scale bar denotes 20 µm. **e** Fluorescent intensity of C6/C-NPs with/without ultrasound treatment was recorded along a straight line from the tumour surface to the interior

In vivo imaging was conducted after injecting DiR (1,1-dioctadecyl-3,3,3,3-tetramethy lindotricarbocyaineiodide)-loaded C-NPs or free DiR solution intravenously into A549 tumour nude mice at 1, 4, 12, and 24 h. Whole body scans were acquired and the photographs were analysed using the IVIS Lumina II system (Caliper Life Sciences, Waltham, MA, USA; Fig. 8a). At 1 h, the fluorescence signals of DiR were maximum for both the DiR/C-NPs and free DiR groups, indicating the active targeting ability of C-NPs and quick distribution of free DiR. However, the DiR signals weakened more quickly in the free DiR group than in the DiR/C-NPs with time, due to the prolonged circulation and active targeting of the CS-based nanoparticles. The free DiR was metabolised quickly. Twenty-four hours following intravenous injection, the mice were sacrificed and their main organs were dissected for fluorometric analysis (Fig. 8b, c). Compared to the results of in vivo biodistribution images at 24 h, C-NPs exhibited stronger fluorescence intensity. This was especially pronounced in tumour tissues, rather than the abdominal organs, according to the ex vivo fluorescence intensity results. The findings might be attributed to the abundant blood flow around the abdomen that facilitated drug delivery. The tumour tissues exhibited the maximum accumulation in the DiR/C-NPs group. Accumulation was nearly 2.9-fold higher than that in the free DiR group, and the liver fluorescence intensity was nearly 2.5-fold stronger than that of the free DiR group.

### Ex vivo tumour penetration

To estimate whether the ultrasound treatment could endow the redox/ultrasound-responsive self-destructive C-NPs with stronger tumour-penetrating ability, intact A549 solid tumours were dissected and incubated with C6/C-NPs with/without SDT treatment followed by preparation of frozen sections. As depicted in Fig. 8d, ultrasound promoted deeper C6 penetration into A549 solid tumours by accelerating the blood flow in tumour vessels. In sections acquired without SDT treatment, C6 fluorescence was mainly observed at the surface of tumour with a penetration depth of only 60.1 µm. In contrast, the depth of C6 penetration in SDT-treated tumour was 231.3 µm from the outer layers to the interior. Penetration was quantified using Image J software. Two lines were drawn from the surface of tumour slices to the inner layers. The fluorescent intensity along the lines was plotted at different distances from the outer layer of the tumour (Fig. 8e). The signal attenuation trends of C6 in C-NPs were consistent with the fluorescent results, suggesting the penetration promoting effect of ultrasound in tumour cells.

### Anti‐tumour efficiency

**Fig. 9:**
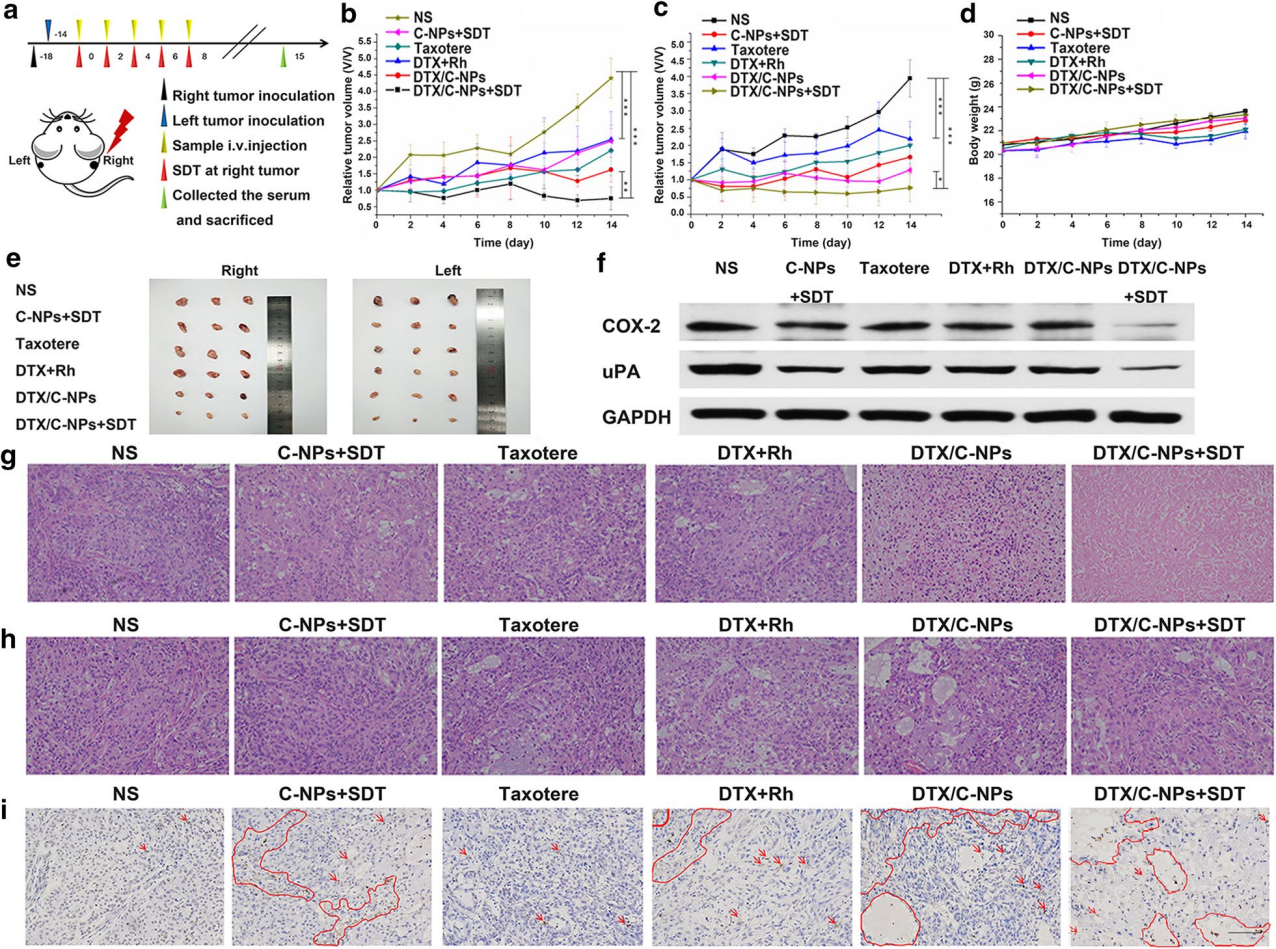
In vivo anti-tumour effect in a bilateral A549 mouse model. **a** Overall schedule of in vivo tumour inhibition study. (**b**−**d**) RTV curves of ‘R’ (**b**) and ‘L’ (**c**) tumours and body weight curves (**d**). **e** Photographs of ‘R’ and ‘L’ tumours after autopsy. **f** Western blot result of expression of COX-2 and uPA proteins after treatment with different solutions at day 15. **g** and **h** H&E staining images of ‘R’ (**g**) and ‘L’ (**h**) tumour tissues after the treatment with NS, C-NPs (SDT+), Taxotere®, DTX + Rh, DTX/C-NPs (SDT + or SDT-). Scale bar denotes 100 µm. **i** TUNEL results of ‘R’ tumours from all bilateral tumour-bearing mice treated with NS, C-NPs (SDT+), Taxotere®, DTX + Rh and DTX/C-NPs (SDT + or SDT-). Scale bar denotes, 100 µm. **p < 0.01, ***p < 0.001

The in vivo anti-tumour effects of DTX/C-NPs in a bilateral A549 tumour-bearing mouse model were evaluated (Fig. 9a). The relative tumour volume (RTV) of bilateral tumour-bearing mice (‘Right (R)’ for in situ tumour and ‘Left (L)’ for distal tumour) was measured. Body weight changes were also recorded (Fig. 9b, c and d). The tumour suppression effect was similar between ‘R’ and ‘L’ tumours. DTX/C-NPs with/without SDT groups displayed significant decrease in RTV after 14 days compared with the control group (NS) with an RTV of 4.40, 3.94, 1.62, 1.28, 0.74 and 0.77 for NS (R), NS (L), DTX/C-NPs (R), DTX/C-NPs (L), DTX/C-NPs + SDT (R) and DTX/C-NPs + SDT (L), respectively. In contrast, the C-NP groups exhibited considerably lower RTV compared with the Taxotere® and DTX + Rh groups, especially in the ‘L’ tumours. This finding probably contributed to the smaller size of distal tumour (Fig. 9e). Subsequently, the body weights of mice, except for the Taxotere® and DTX + Rh groups, exhibited no significant change compared with the NS group. The curves of Taxotere® and DTX + Rh showed that mice gained the minimum body weight on day 10, but regained the loss after treatment stopped. Therefore, the free drug was cumulatively toxic to mice but the drug could be metabolised quickly when its’ administration was stopped.

Furthermore, the expression of cyclooxygenase-2 (COX-2) and urokinase-type plasminogen activator (uPA) proteins in ‘L’ tumour tissues was evaluated via western blot assay to observe these tumour metastasis-related indicators in tissue homogenates (Fig. 9f). The expression of both COX-2 and uPA proteins was decreased in the C-NP + SDT and DTX/C-NPs + SDT groups compared with the NS group. The findings indicated the good anti-metastasis effect of SDT and chemo-SDT treatments. Hematoxylin and eosin staining (H&E) staining of sections revealed no obvious pathological damage to the main organs (Additional file 1: Fig. S9). Additionally, all ‘R’ and ‘L’ tumour were stained with H&E. A pro-apoptotic effect was readily evident with a small amount of blue staining nuclei in cells in DTX/C-NPs and DTX/C-NPs + SDT groups. In addition, the C-NPs + SDT group also displayed reduced nuclei compared with the NS group. However, the pro-apoptotic effect of SDT and combined therapy for ‘L’ tumour was less distinct than the ‘R’ results (Fig. 9g and h). TdT-mediated dUTP Nick-End Labelling (TUNEL) assay showed similar results for ‘R’ and ‘L’ tumour (Fig. 9i and Additional file 1: Fig. S10).

In TUNEL sections, the apoptotic cells stained yellow or pale brown colour as indicated by red arrows or curves in Fig. 9i and Additional file 1: Fig. S10. Normal cells contained blue stained nuclei. The NS groups showed negligible tumour apoptosis, while SDT induced pronounced cell apoptosis apparent by the larger brown areas in the slices. The combined therapy of DTX/C-NPs + SDT was the most effective in killing tumour cells, as seen by the large number of brown vacuoles with several apoptotic nuclei. The DTX/C-NPs group showed low efficacy of apoptosis. Tumour tissues in the DTX + Rh group exhibited more apoptosis than the Taxotere® group, due to the low production of ROS by Rh.

### Serum composition

**Fig. 10:**
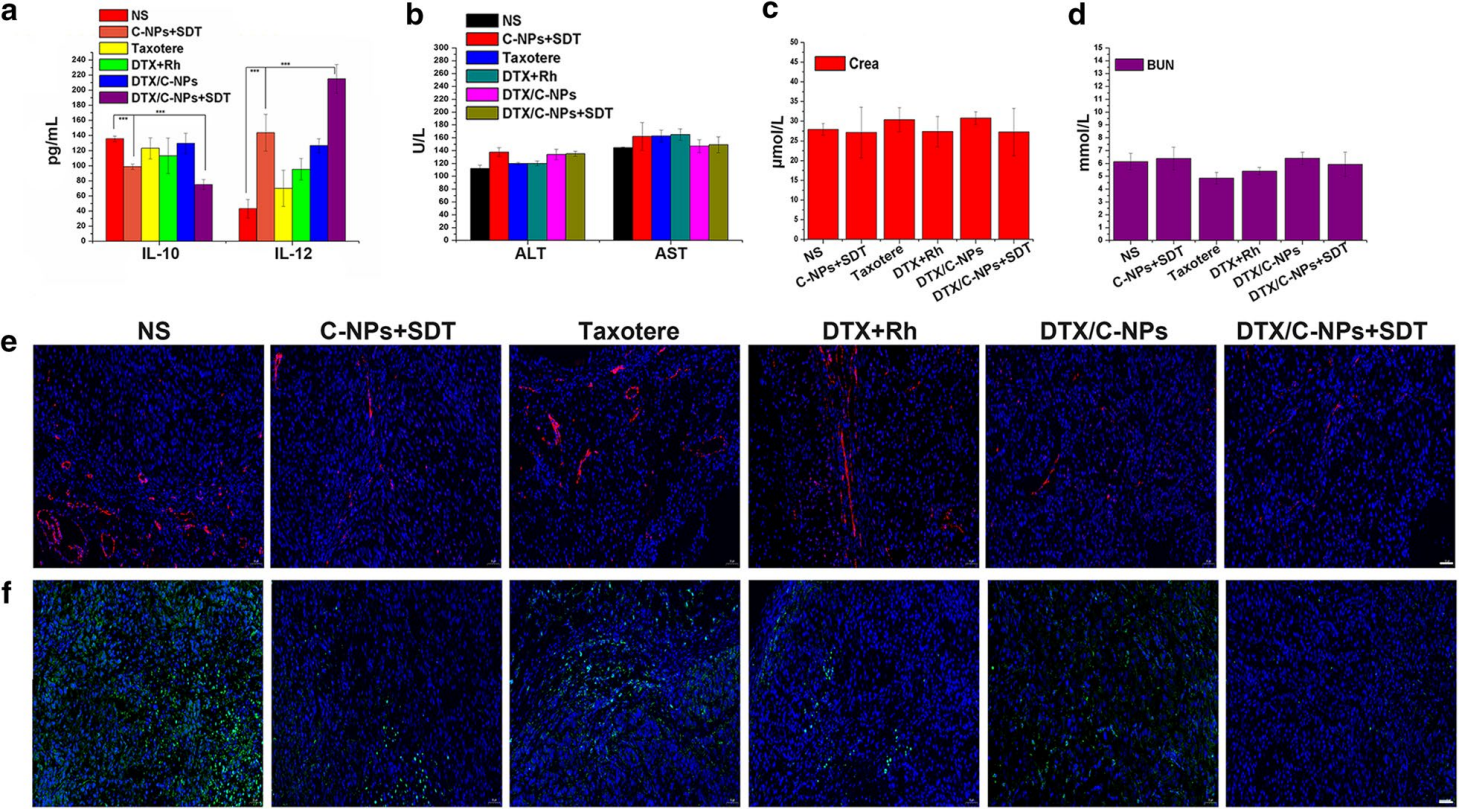
Serum composition and immunofluorescence results. **a**–**d** Measurements of IL-10, IL-12 (**a**), ALT, AST (**b**), CREA (**c**) and BUN (**d**) in mouse serum after treating with NS, C-NPs + SDT, Taxotere®, DTX + Rh, DTX/C-NPs and DTX/C-NPs + SDT. ***p < 0.001. **e** and **f** Fluorescence images of anti-CD31-stained blood vessels (red fluorescence; **e**), anti-CD206-stained M2 type macrophages (green fluorescence; **f**) and 4’,6-diamidino-2-phenylindole (DAPI)-stained nuclei, respectively, by slices collected from ‘L’ tumours after the different treatments. Scale bar denotes, 50 µm

Considering the RTV and H&E staining results for ‘R’ and ‘L’ tumours, we speculated that the immune system of mice might be activated during the course of administration after SDT treatment. To explore this, interleukin-10 (IL-10) and IL-12 were measured in serum using an ELISA kit to evaluate the generation of M2 and M1 macrophage subtypes in the presence of pro/anti-inflammatory cytokines after different treatments [40]. The IL-10 content decreased after SDT treatment with C-NPs or DTX/C-NPs. The content of IL-12 increased (Fig. 10a). The findings indicated that SDT could elevate the expression of M1 macrophages while decreasing the M2 type, which could enhance the immune capacity of macrophages of the DTX/C-NPs group upon the use of ultrasound. In addition, the serum biochemical indicators alanine aminotransferase (ALT), aspartate aminotransferase (AST), creatinine (CREA) and blood urea nitrogen (BUN) were detected (Fig. 10b, c and d). Their contents did not differ significantly in the different treatment groups, indicating the safety of DTX/C-NPs + SDT administration in the heart, liver and kidney.

### Immunocytochemical staining

Based on the disparate expression of MMP9 and VEGFA proteins in Fig. 5b, immunofluorescence was used to examine ‘L’ tumour tissues with the anti-CD31 marker to clarify whether angiogenesis in tumours was inhibited or not after SDT treatment (Fig. 10e). Endothelial cells in platelets, which were apparent as red fluorescence in the sections, disappeared after C-NPs + SDT, DTX/C-NPs and DTX/C-NPs + SDT treatments compared to the NS group. The findings indicated the good vascular inhibition by DTX/C-NPs and SDT. Thus, angiogenesis was inhibited after DTX/C-NPs + SDT treatment, but not through the VEGFA pathway. In addition, M2 macrophages in tumour tissues were stained with the anti-CD206 marker and imaged. A histogram of IL-10 values revealed that fewer M2 macrophages was produced after SDT treatment compared with the other groups, as evident by increased green fluorescence.

## Conclusions

An active tumour-targeting nanoplatform based on DTX/C-NPs and SDT was constructed. The platform enabled combined chemo-SDT. Using LA to formulate the intermolecular disulfide bonds after crosslinking, together with Rh as the novel discovered SS enabled, redox/ultrasound-responsive property of the nanoparticles. DTX/C-NPs enhanced the solubility and cellular uptake ability of DTX, and also displayed reduced toxicity to normal cells. Combined with the innate ROS-generating ability and SDT effect of Rh, DTX/C-NPs could generate ROS rapidly and continuously, which significantly inhibited tumour metastasis. Furthermore, DTX/C-NPs also showed good targeting ability to the Golgi apparatus and mitochondria, leading to the destruction of their structures after SDT treatment. Due to the encapsulation of DTX, the nanoparticles effectively induced changes in microtubule morphology and blocked the mitotic cycle of A549 cancer cells. Benefiting from the penetration promoting effect of ultrasound treatment, DTX/C-NPs penetrated more deeply into tumour tissues, with better and safer suppression of tumour growth compared with non-ultrasonically treated tumours. The immune system of mice was obviously activated after SDT by expressing fewer M2 type macrophages while improving the formation of M1 type macrophages. Angiogenesis of tumour vessels was inhibited at the same time. All preparations were safe for mice.

## Supplementary Information

**Additional file 1: Figure S1.** The synthesis steps of CS-ADH-Rh-LA conjugates. **Figure S2** The characterization of CS-ADH-Rh-LA. The ^1^H NMR (A) and ^13^ C NMR (B) spectra of CS, CS-ADH-Rh and CS-ADH-Rh-LA copolymers. (C) The FT-IR spectra of CS, CS-ADH-Rh and CS-ADH-Rh-LA copolymers. **Figure S3** The DLS result of C-NPs after incubating in 20 µM DTT solution for 24 h. **Figure S4** Size changes of C-NPs and NC-NPs in 10% FBS solution within 6 days. **Figure S5** Transcellular transport assay of C6/C-NPs in A549 cells. Scar bar, 100 µm. **Figure S6** The cell viability of A549 cells after incubating with different concentrations of DTX. **Figure S7** Cytotoxicity result of CS-ADH-LA with different concentrations. **Figure S8** The CLSM images of ROS production in A549 cells after incubating with different solutions with/without SDT treatment. Scar bar, 100 µm. **Figure S9** The H&E staining results of heart, liver, spleen, lung and kidney after incubating with NS, C-NPs with SDT, Taxotere®, DTX with Rh, DTX/C-NPs and DTX/C-NPs with SDT. Scar bar, 100 µm. **Figure S10** TUNEL images of Left tumor tissues from bilateral tumor bearing mice treated with NS, C-NPs + SDT, Taxotere®, DTX + Rh, DTX/C-NPs and DTX/C-NPs + SDT. Scar bar, 100 µm.

**Additional file 2.** Described the experimental methods.

## Data Availability

All data generated or analysed during this are included in this published article.

## References

[1] Hirsch FR, Scagliotti GV, Mulshine JL, Kwon R, Curran WJ, Wu Y-L, Paz-Ares L (2017). Lung cancer: current therapies and new targeted treatments. Lancet.

[2] Califano R, Gomes F, Ackermann CJ, Rafee S, Tsakonas G, Ekman S (2020). Immune checkpoint blockade for non-small cell lung cancer: what is the role in the special populations?. Eur J Cancer.

[3] Arbour KC, Riely GJ (2019). Systemic therapy for locally advanced and metastatic non-small cell lung cancer: a review. JAMA.

[4] Vijayaraghavalu S, Gao Y, Rahman MT, Rozic R, Sharifi N, Midura RJ, Labhasetwar V (2020). Synergistic combination treatment to break cross talk between cancer cells and bone cells to inhibit progression of bone metastasis. Biomaterials.

[5] Liu M, Khan AR, Ji J, Lin G, Zhao X, Zhai G (2018). Crosslinked self-assembled nanoparticles for chemo-sonodynamic combination therapy favoring antitumor, antimetastasis management and immune responses. J Control Release.

[6] Zhang D, Zhang J, Li Q, Song A, Li Z, Luan Y (2019). Cold to hot: rational design of a minimalist multifunctional photo-immunotherapy nanoplatform toward boosting immunotherapy capability. ACS Appl Mater Interfaces.

[7] Liang S, Deng X, Chang Y, Sun C, Shao S, Xie Z, Xiao X, Ma P, Zhang H, Cheng Z, Lin J (2019). Intelligent hollow Pt-CuS janus architecture for synergistic catalysis-enhanced sonodynamic and photothermal cancer therapy. Nano Lett.

[8] Zheng X, Liu W, Ge J, Jia Q, Nan F, Ding Y, Wu J, Zhang W, Lee CS, Wang P (2019). Biodegradable natural product-based nanoparticles for near-infrared fluorescence imaging-guided sonodynamic therapy. ACS Appl Mater Interfaces.

[9] Wu W, Mao D, Hu F, Xu S, Chen C, Zhang CJ, Cheng X, Yuan Y, Ding D, Kong D, Liu B (2017). A highly efficient and photostable photosensitizer with near-infrared aggregation-induced emission for image-guided photodynamic anticancer therapy. Adv Mater.

[10] Song D, Yue W, Li Z, Li J, Zhao J, Zhang N (2014). Study of the mechanism of sonodynamic therapy in a rat glioma model. Onco Targets Ther.

[11] Wang P, Zhou F, Guan K, Wang Y, Fu X, Yang Y, Yin X, Song G, Zhang X-B, Tan W (2020). In vivo therapeutic response monitoring by a self-reporting upconverting covalent organic framework nanoplatform. Chem Sci.

[12] Zhang Q, Wang N, Ma M, Luo Y, Chen H (2019). Transferrin receptor-mediated sequential intercellular nanoparticles relay for tumor deep penetration and sonodynamic therapy. Adv Ther.

[13] Huang J, Liu F, Han X, Zhang L, Hu Z, Jiang Q, Wang Z, Ran H, Wang D, Li P (2018). Nanosonosensitizers for highly efficient sonodynamic cancer theranostics. Theranostics.

[14] Yang ZL, Tian W, Wang Q, Zhao Y, Zhang YL, Tian Y, Tang YX, Wang SJ, Liu Y, Ni QQ, Lu GM, Teng ZG, Zhang LJ (2018). Oxygen-evolving mesoporous organosilica coated prussian blue nanoplatform for highly efficient photodynamic therapy of tumors. Adv Sci (Weinh).

[15] Hu C, Huang HW, Chen F, Zhang YH, Yu H, Ma TY (2020). Coupling piezocatalysis and photocatalysis in Bi4NbO8X (X = Cl, Br) polar single crystals. Adv Func Mater..

[16] Singh J, Jadhav S, Avasthi S, Sen P (2020). Designing photocatalytic nanostructured antibacterial surfaces: why is black silica better than black silicon?. ACS Appl Mater Interfaces.

[17] Wu M, Ding Y, Li L (2019). Recent progress in the augmentation of reactive species with nanoplatforms for cancer therapy. Nanoscale.

[18] Qian X, Zheng Y, Chen Y (2016). Micro/nanoparticle-augmented sonodynamic therapy (SDT): breaking the depth shallow of photoactivation. Adv Mater.

[19] Li-Weber M (2013). Targeting apoptosis pathways in cancer by Chinese medicine. Cancer Lett.

[20] Yang L, Lin S, Kang Y, Xiang Y, Xu L, Li J, Dai X, Liang G, Huang X, Zhao C (2019). Rhein sensitizes human pancreatic cancer cells to EGFR inhibitors by inhibiting STAT3 pathway. J Exp Clin Cancer Res.

[21] Wang A, Jiang H, Liu Y, Chen J, Zhou X, Zhao C, Chen X, Lin M (2020). Rhein induces liver cancer cells apoptosis via activating ROS-dependent JNK/Jun/caspase-3 signaling pathway. J Cancer.

[22] Hu L, Cui R, Liu H, Wang F. Emodin and rhein decrease levels of hypoxia-inducible factor-1α in human pancreatic cancer cells and attenuate cancer cachexia in athymic mice carrying these cells. Oncotarget. 2017;8. 10.18632/oncotarget.21330. 10.18632/oncotarget.21330 PMC5675689 29152137

[23] Jardim KV, Siqueira JLN, Bao SN, Sousa MH, Parize AL (2020). The role of the lecithin addition in the properties and cytotoxic activity of chitosan and chondroitin sulfate nanoparticles containing curcumin. Carbohydr Polym.

[24] Xie C, Wang Q, Ying R, Wang Y, Wang Z, Huang M (2020). Binding a chondroitin sulfate-based nanocomplex with kappa-carrageenan to enhance the stability of anthocyanins. Food Hydrocolloid.

[25] Zhang X, Ma Y, Ma L, Zu M, Song H, Xiao B (2019). Oral administration of chondroitin sulfate-functionalized nanoparticles for colonic macrophage-targeted drug delivery. Carbohydr Polym.

[26] Shi X, Yang X, Liu M, Wang R, Qiu N, Liu Y, Yang H, Ji J, Zhai G (2021). Chondroitin sulfate-based nanoparticles for enhanced chemo-photodynamic therapy overcoming multidrug resistance and lung metastasis of breast cancer. Carbohydr Polym.

[27] Huang L, Wang Y, Ling X, Chaurasiya B, Yang C, Du Y, Tu J, Xiong Y, Sun C (2017). Efficient delivery of paclitaxel into ASGPR over-expressed cancer cells using reversibly stabilized multifunctional pullulan nanoparticles. Carbohydr Polym.

[28] Liao C, Dai X, Chen Y, Liu J, Yao Y, Zhang S (2019). Biogenic (R)-(+)-lipoic acid only constructed cross-linked vesicles with synergistic anticancer potency. Adv Func Mater.

[29] Huang J, Wang L, Zhao P, Xiang F, Liu J, Zhang S (2018). Nanocopper-doped cross-linked lipoic acid nanoparticles for morphology-dependent intracellular catalysis. ACS Catalysis.

[30] Yang Y, Zhu H, Wang J, Fang Q, Peng Z (2018). Enzymatically disulfide-crosslinked chitosan/hyaluronic acid layer-by-layer self-assembled microcapsules for redox-responsive controlled release of protein. ACS Appl Mater Interfaces.

[31] Li A, Zhang D (2016). Synthesis and characterization of cleavable core-cross-linked micelles based on amphiphilic block copolypeptoids as smart drug carriers. Biomacromol.

[32] Li L, Sun W, Zhang Z, Huang Y (2016). Time-staggered delivery of docetaxel and H1-S6A,F8A peptide for sequential dual-strike chemotherapy through tumor priming and nuclear targeting. J Control Release.

[33] Zhou H, Lv S, Zhang D, Deng M, Zhang X, Tang Z, Chen X (2018). A polypeptide based podophyllotoxin conjugate for the treatment of multi drug resistant breast cancer with enhanced efficiency and minimal toxicity. Acta Biomater.

[34] Trinh T, Liao C, Toader V, Barlog M, Bazzi HS, Li J, Sleiman HF (2018). DNA-imprinted polymer nanoparticles with monodispersity and prescribed DNA-strand patterns. Nat Chem.

[35] Yao C, Wang W, Wang P, Zhao M, Li X, Zhang F (2018). Near-infrared upconversion mesoporous cerium oxide hollow biophotocatalyst for concurrent pH-/H2 O2 -responsive O2 -evolving synergetic cancer therapy. Adv Mater..

[36] Gao M, Yi J, Zhu J, Minikes AM, Monian P, Thompson CB, Jiang X (2019). Role of mitochondria in ferroptosis. Mol Cell.

[37] Ma T, Hou Y, Zeng J, Liu C, Zhang P, Jing L, Shangguan D, Gao M (2018). Dual-ratiometric target-triggered fluorescent probe for simultaneous quantitative visualization of tumor microenvironment protease activity and pH in vivo. J Am Chem Soc.

[38] Gao H, Xie W, Yang C, Xu J, Li J, Wang H, Chen X, Huang CF (2018). NRAMP2, a trans-Golgi network-localized manganese transporter, is required for Arabidopsis root growth under manganese deficiency. New Phytol.

[39] Eisenberg-Lerner A, Benyair R, Hizkiahou N, Nudel N, Maor R, Kramer MP, Shmueli MD, Zigdon I, Cherniavsky Lev M, Ulman A, Sagiv JY, Dayan M, Dassa B, Rosenwald M, Shachar I, Li J, Wang Y, Dezorella N, Khan S, Porat Z, Shimoni E, Avinoam O, Merbl Y (2020). Golgi organization is regulated by proteasomal degradation. Nat Commun.

[40] Larionova I, Kazakova E, Patysheva M, Kzhyshkowska J. Transcriptional. Epigenetic and Metabolic Programming of Tumor-Associated Macrophages. Cancers (Basel). 2020;12. doi:10.3390/cancers12061411. 10.3390/cancers12061411 PMC7352439 32486098

