# Supplementary material for: A sonosensitiser‐based polymeric nanoplatform for chemo‐sonodynamic combination therapy of lung cancer
Source: J Nanobiotechnology. 2021 Feb 25;19:57. doi: 10.1186/s12951-021-00804-9 (PMC7905889; doi:10.1186/s12951-021-00804-9)
Supplement: Supplementary file 1 — Additional file 1: Figure S1. The synthesis steps of CS-ADH-Rh-LA conjugates. Figure S2 The characterization of CS-ADH-Rh-LA. The 1H NMR (A) and 13 C NMR (B) spectra of CS, CS-ADH-Rh and CS-ADH-Rh-LA copolymers. (C) The FT-IR spectra of CS, CS-ADH-Rh and CS-ADH-Rh-LA copolymers. Figure S3 The DLS result of C-NPs after incubating in 20 µM DTT solution for 24 h. Figure S4 Size changes of C-NPs and NC-NPs in 10% FBS solution within 6 days. Figure S5 Transcellular transport assay of C6/C-NPs in A549 cells. Scar bar, 100 µm. Figure S6 The cell viability of A549 cells after incubating with different concentrations of DTX. Figure S7 Cytotoxicity result of CS-ADH-LA with different concentrations. Figure S8 The CLSM images of ROS production in A549 cells after incubating with different solutions with/without SDT treatment. Scar bar, 100 µm. Figure S9 The H&E staining results of heart, liver, spleen, lung and kidney after incubating with NS, C-NPs with SDT, Taxotere®, DTX with Rh, DTX/C-NPs and DTX/C-NPs with SDT. Scar bar, 100 µm. Figure S10 TUNEL images of Left tumor tissues from bilateral tumor bearing mice treated with NS, C-NPs + SDT, Taxotere®, DTX + Rh, DTX/C-NPs and DTX/C-NPs + SDT. Scar bar, 100 µm. [file 12951_2021_804_MOESM1_ESM.docx]

Curves and table Section


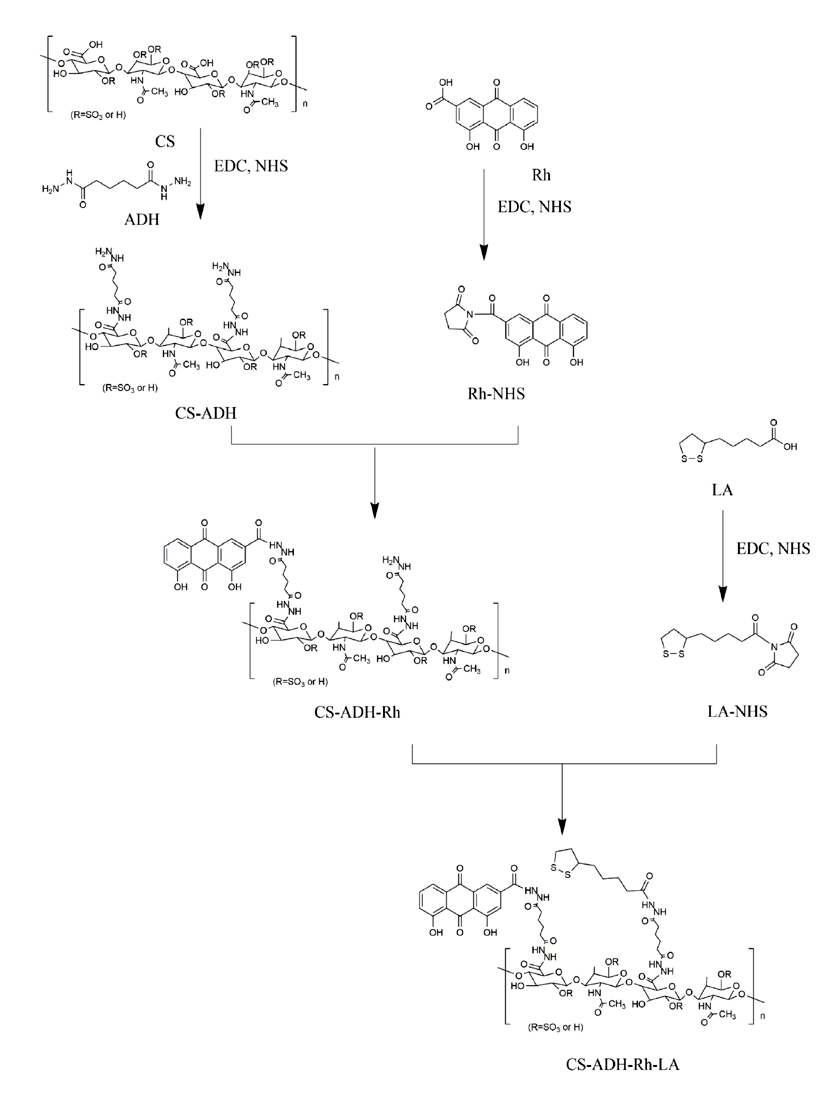


**Fig. S1** The synthesis steps of CS-ADH-Rh-LA conjugates.


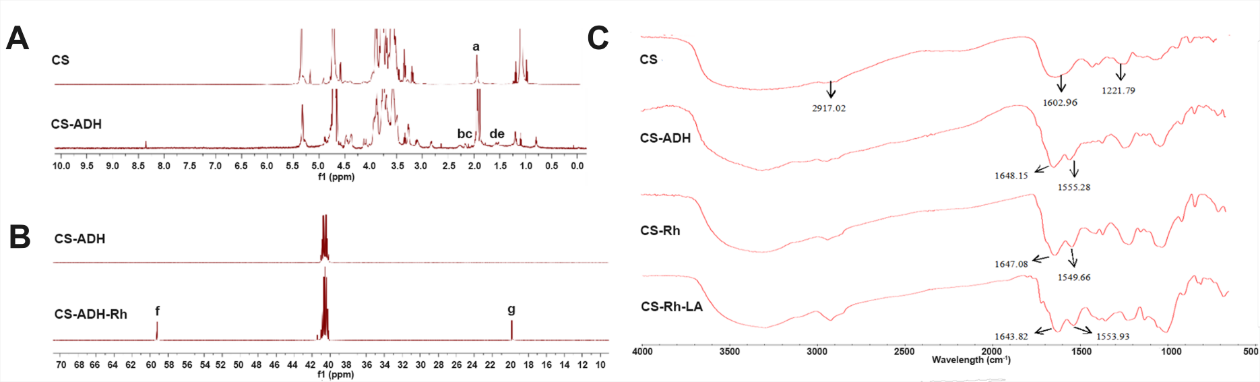


**Fig. S2** The characterization of CS-ADH-Rh-LA. The ^1^H NMR (A) and ^13^C NMR (B) spectra of CS, CS-ADH-Rh and CS-ADH-Rh-LA copolymers. (C) The FT-IR spectra of CS, CS-ADH-Rh and CS-ADH-Rh-LA copolymers.


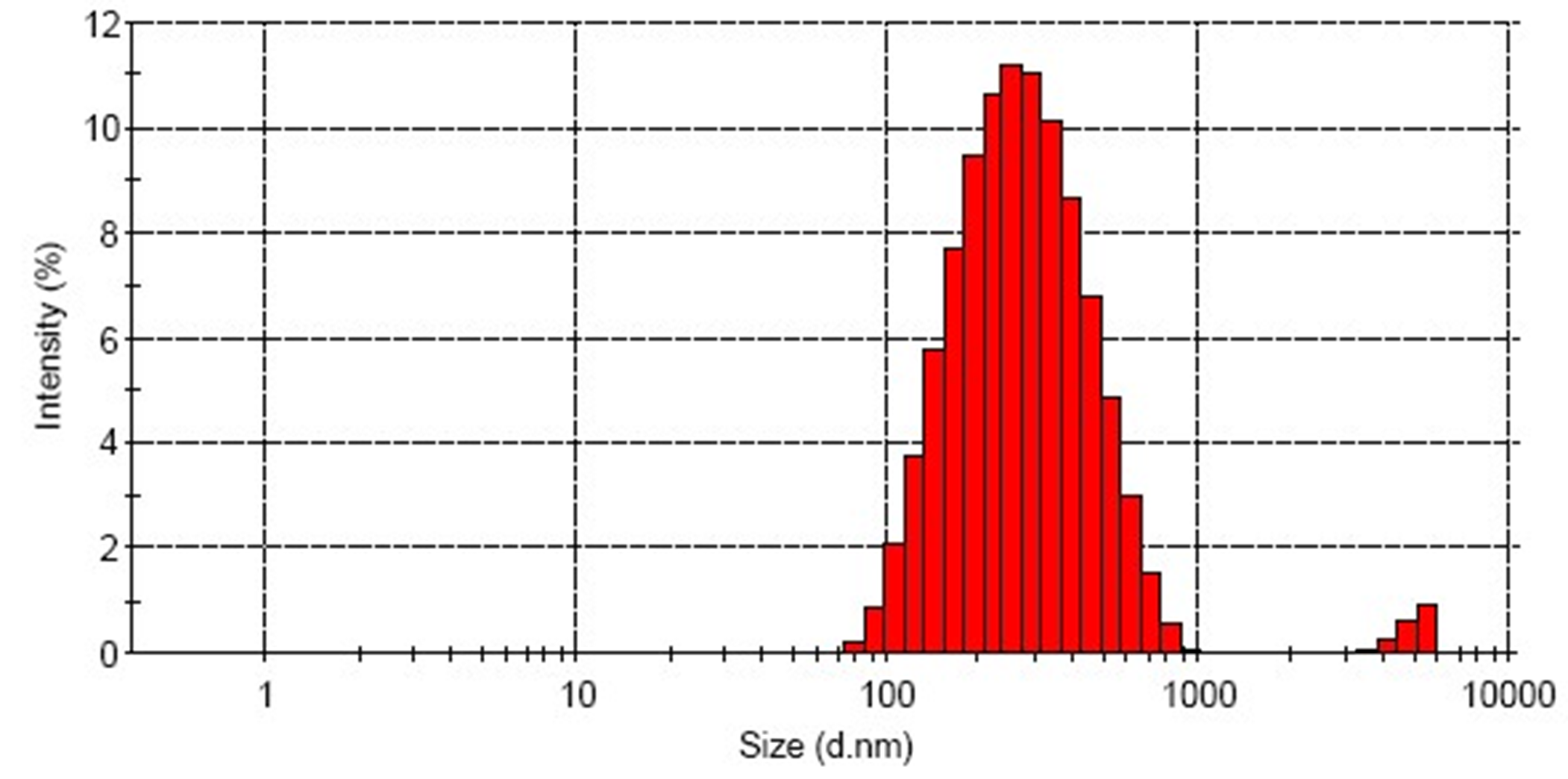


**Fig. S3** The DLS result of C-NPs after incubating in 20 μM DTT solution for 24 h.


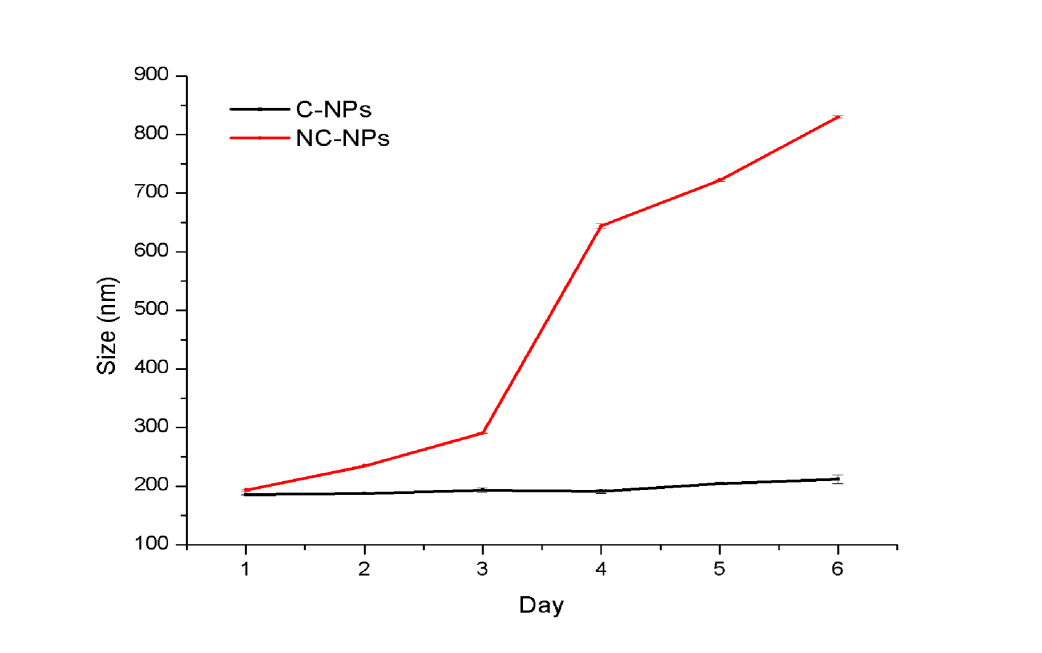


**Fig. S4** Size changes of C-NPs and NC-NPs in 10% FBS solution within 6 days.


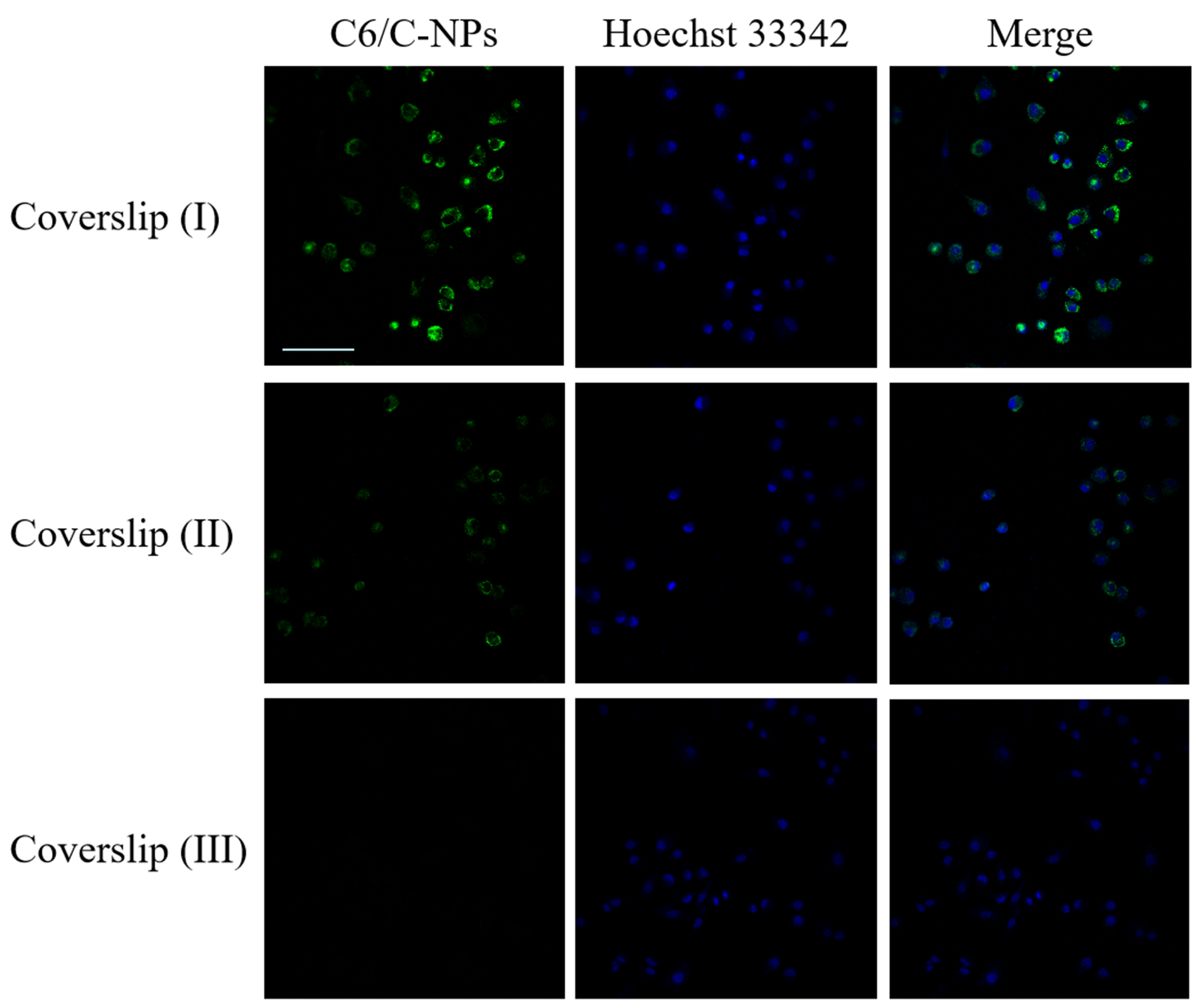


**Fig. S5** Transcellular transport assay of C6/C-NPs in A549 cells. Scar bar, 100 μm.


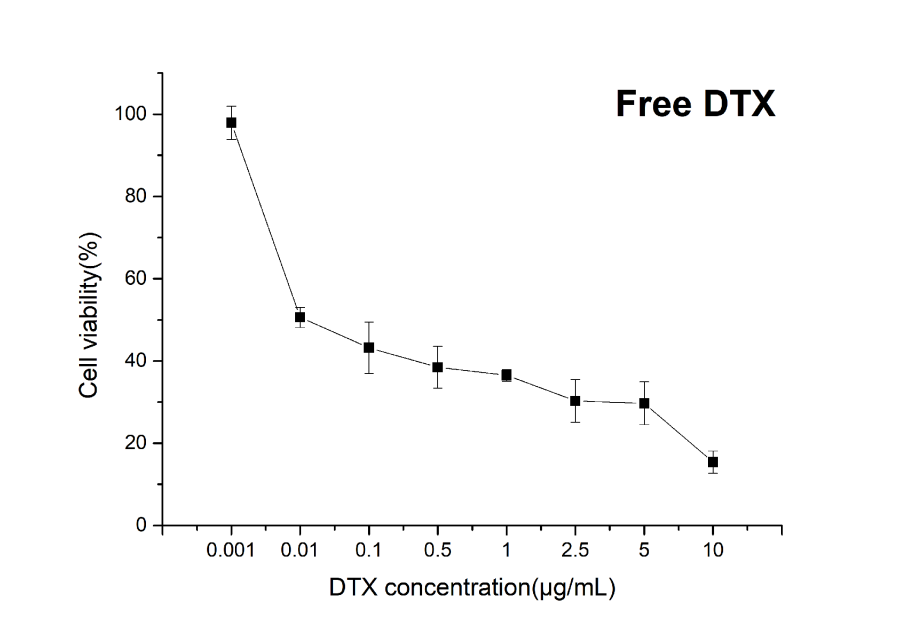


**Fig. S6** The cell viability of A549 cells after incubating with different concentrations of DTX.





**Fig. S7** Cytotoxicity result of CS-ADH-LA with different concentrations.


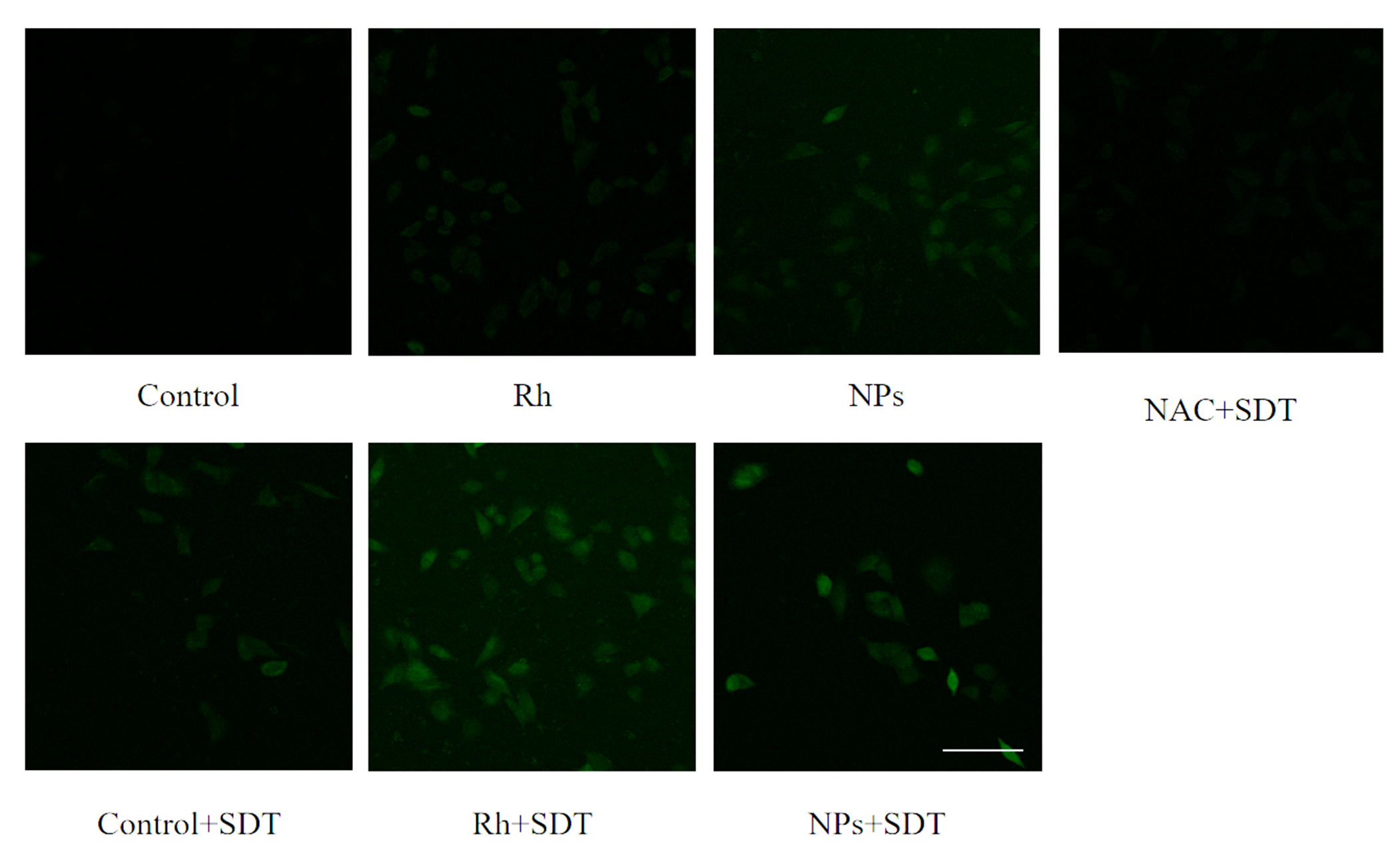


**Fig. S8** The CLSM images of ROS production in A549 cells after incubating with different solutions with/without SDT treatment. Scar bar, 100 μm.


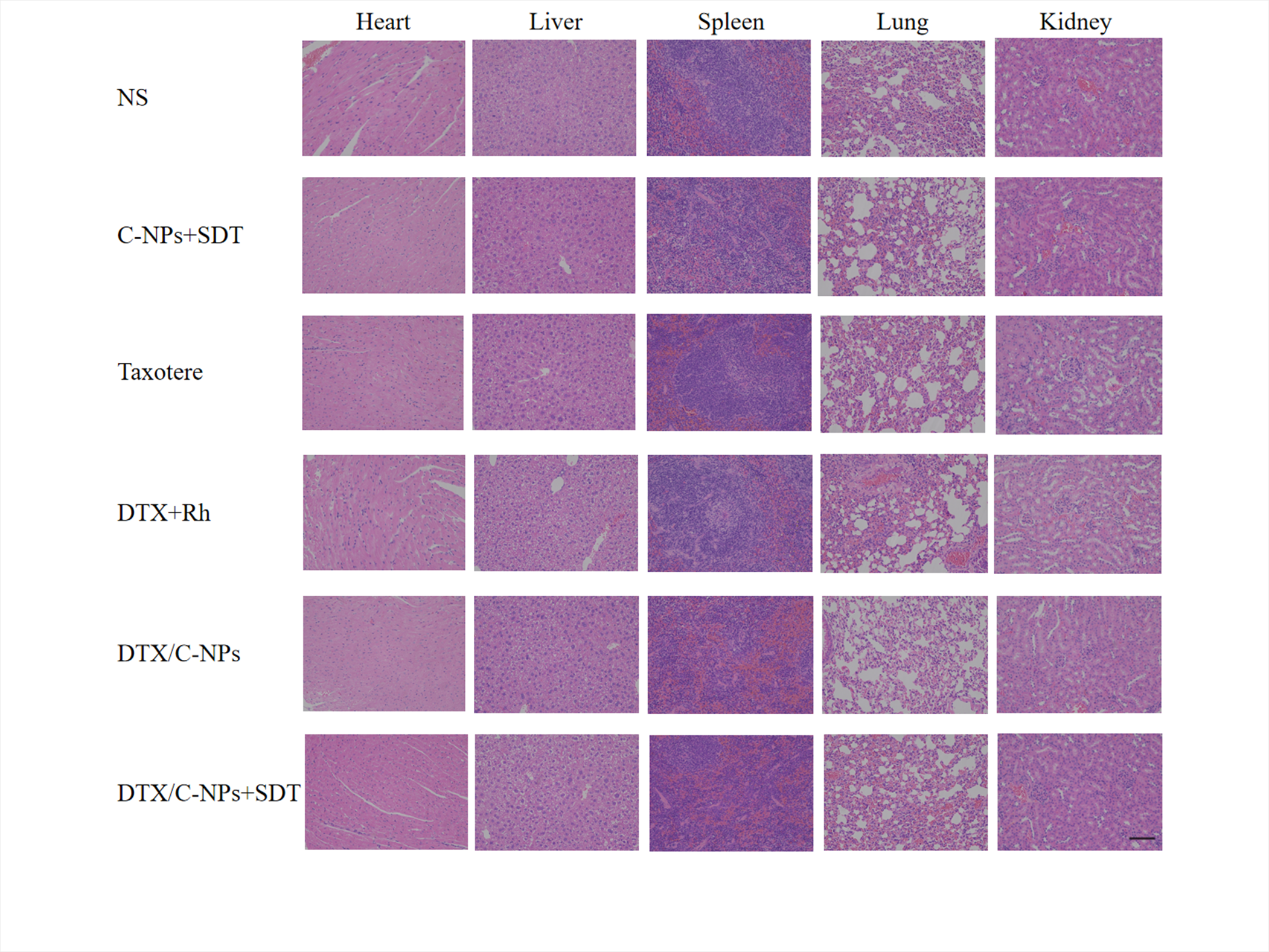


**Fig. S9** The H&E staining results of heart, liver, spleen, lung and kidney after incubating with NS, C-NPs with SDT, Taxotere, DTX with Rh, DTX/C-NPs and DTX/C-NPs with SDT. Scar bar, 100 μm.


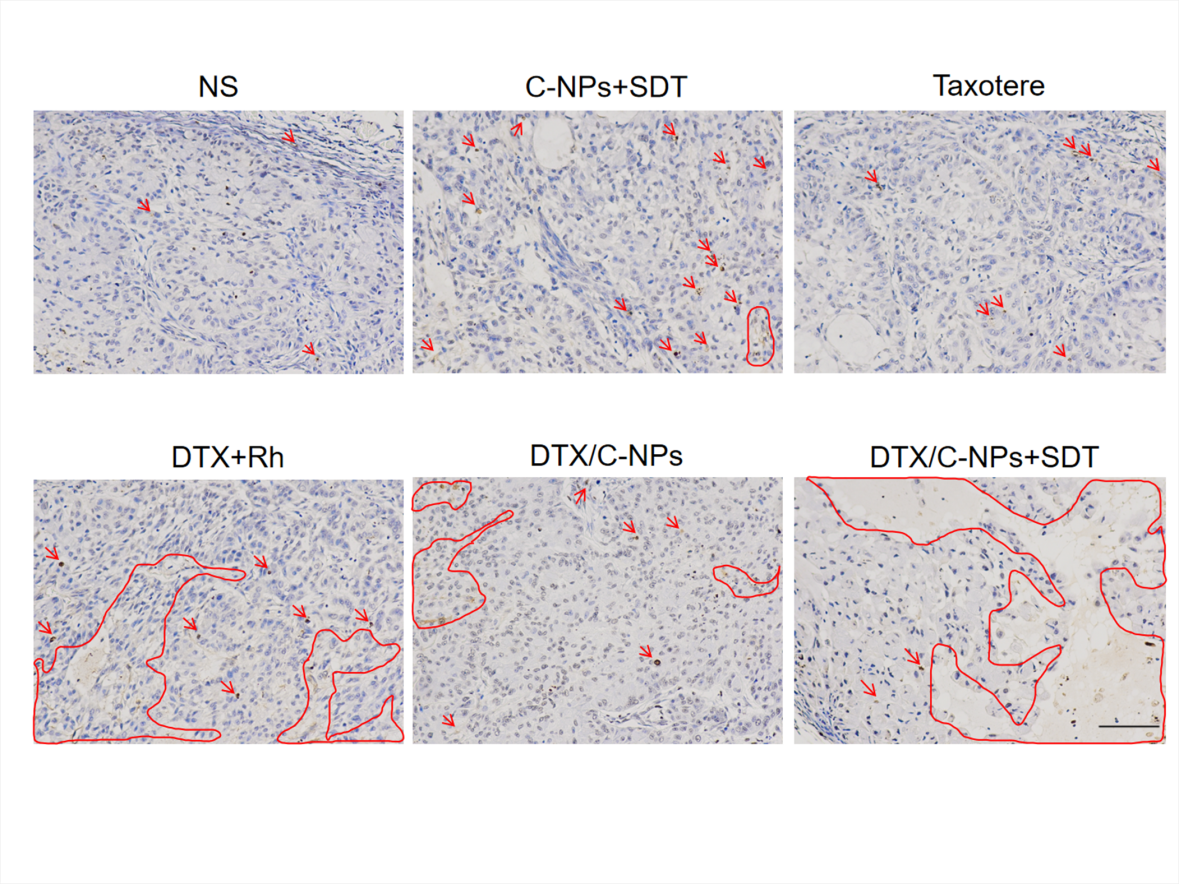


**Fig. S10** TUNEL images of Left tumor tissues from bilateral tumor bearing mice treated with NS, C-NPs +SDT, Taxotere, DTX+Rh, DTX/C-NPs and DTX/C-NPs+SDT. Scar bar, 100 μm.
