# Supplementary material for: A sonosensitiser‐based polymeric nanoplatform for chemo‐sonodynamic combination therapy of lung cancer
Source: J Nanobiotechnology. 2021 Feb 25;19:57. doi: 10.1186/s12951-021-00804-9 (PMC7905889; doi:10.1186/s12951-021-00804-9)
Supplement: Supplementary file 2 — Additional file 2. Described the experimental methods. [file 12951_2021_804_MOESM2_ESM.docx]

Supporting Information

**A** **Sonosensitiser-based Polymeric Nanoplatform for Chemo-sonodynamic Combination Therapy of Lung Cancer**

Yanan Zhang^1^, Abdur Rauf Khan^1^, Xiaoye Yang^1^, Yikang Shi^2^, Xiaogang Zhao^3^* and Guangxi Zhai^1^*

E-mail: [zhaoxiaogang@sdu.edu.cn](mailto:zhaoxiaogang@sdu.edu.cn)；mail: professorgxzhai@126.com

^1^ Department of Pharmaceutics, Key Laboratory of Chemical Biology (Ministry of Education), School of Pharmaceutical Sciences, Shandong University, Jinan, 250012, PR China

^2^ National Glycoengineering Research Center, Shandong University, Jinan 250012, China.

^3^ Department of Thoracic Surgery, The Second Hospital of Shandong University, Jinan, Shandong 250033, China.

Tel.: (86) 531-88382015

**Experimental**

**Materials, equipment, cells and mice**

CS (Mw: 10 kDa) was purchased from Xuzhou Jiangda Biotechnology Co., Ltd. (Xuzhou, China). DTX was purchased from Heowns Biochemical Technology Co. (Tianjin, China). ADH (>99.0%), 1-ethyl-3-(3-dimethylaminopropyl)- carbodiimide hydrochloride (98%, EDC) and N-hydroxysuccinimide (NHS) were purchased from Aladdin Bio-Chem Technology Co., Ltd. (Shanghai, China). Rh (> 98%) was acquired from MREDA Technologies Co., Ltd. Beijing, China). Dithiothreitol (DTT, 99%) was obtained from Solarbio Science & Technology Co., Ltd. (Beijing, China). LA (98%) was purchased from Tokyo Chemical Industry Co., Ltd. (Tokyo, Japan). Coumarin 6 (C6, 98%), [Cell Counting Kit 8](https://b2b.baidu.com/b2bsearch/jump?url=https://www.912688.com/supply/323066515.html&query=CCK-8&logid=4050804576&srcId=27729&category=%E5%8C%96%E5%B7%A5%E8%83%BD%E6%BA%90;%E5%8C%96%E5%AD%A6%E8%AF%95%E5%89%82;%E5%85%B6%E4%BB%96%E5%8C%96%E5%AD%A6%E8%AF%95%E5%89%82&sv_cr=0&uign=5a6851d1985c0fe126ab3406959afd67&iid=59288da58888137f1455827c57de5d4d&timeSignOri=1577775269&xzhid=28406439&miniId=8469&ii_pos=0&from=b2b_straight&srcid=5103&from_restype=product), and N-acetylcysteine (NAC) were obtained from Sigma-Aldrich (St. Louis, MO, USA). Foetal bovine serum (FBS), Dulbecco’s modified Eagle’s medium (DMEM) and RPMI-1640 medium were obtained from Gibco BRL (Grand Island, NY, USA). All other materials and reagents were analytical grade.

The therapeutic ultrasound device was a model DM-300B medical ultrasonic therapy instrument (Shenzhen Dimip Technology Co., Ltd., Shenzhen, China). The device was used for ultrasound stimulation at 1 MHz using a duty cycle of 40%, with a 10-ms pulse repetition cycle. The acoustic intensity varied from 0.3 to 3.0 W/cm^2^. Considering clinical practice and safety, an acoustic intensity of 1.2 W/cm^2^ was applied. All [parameters](file:///C:/Users/dell/Desktop/Manuscript%20-2.docx#/javascript:;) remained the same throughout all SDT studies.

A549 adenocarcinoma human alveolar basal epithelial cells and H1299 human NSCLC cells were obtained from the Institute of Biochemical and Biotechnological Drugs at Shandong University. The cells were grown in RPMI 1640 and DMEM complete medium, respectively. Each medium was supplemented with 10% FBS, 250 µg/mL amphotericin B, 100 U/mL penicillin G and 100 mg/mL streptomycin. Culture conditions were 37°C and a 5% CO_2_ atmosphere.

Balb/c nude mice (6–8 weeks old) were acquired from Beijing Vital River Laboratory Animal Technology Co. Ltd. (Beijing, China). All animal studies were conducted after approval of the Institutional Animal Care and Use Guides.

**Polymer synthesis**

The final synthesis of CS-ADH-Rh-LA was performed in triplicate by the amide reaction (Fig. S1). First, the amino terminals of ADH were coupled with the carboxyl groups of CS to synthesise CS-ADH as previously described with some modifications [[1](#_ENREF_1)]. Then, 2.0 g EDC and 0.8 g NHS were added to the pre-dissolved CS in distilled water (DW; final CS concentration of 10 mg/mL) 15 min before adding 3.52 g ADH. The reaction proceeded by stirring for 24 h at 25°C. The product was lyophilized before being dialyzed against DW using a membrane with a molecular weight cutoff (MWCO) of 3500 to remove residual agents. ADH was quantified by comparing the integration proportion in the ^1^H-NMR spectra of ADH and CS.

Rh was grafted to CS-ADH using the same method for the synthesis of Rh-lysinate. Rh-CS-ADH was successfully synthesised by a trial-and-error method because of the lack of available methods [[2](#_ENREF_2)]. First, EDC and NHS (5 equiv. / Rh for each) were added to Rh in dimethylsulfoxide (DMSO) for 4 h in a nitrogen atmosphere in the dark. CS-ADH (100 mg) was dissolved in 10 mL of a 1:1 mixed solvent of DW and DMSO. The solution was added to the Rh solution. The proportion of Rh to CS-ADH varied from 1:10 to 3:10. The reaction was continued for 24 h in the dark at 25°C. The sample was then dialyzed (MWCO 3500) against DMSO and DW, and lyophilized. The degree of substitution (DS) of Rh was detected using an ultraviolet-visible (UV-Vis) spectrophotometer at 443 nm.

LA was joined to CS-Rh conjugates through the [amidation](file:///C:/Users/dell/Desktop/Manuscript%20-2.docx#/javascript:;) coupling of LA with ADH [[1](#_ENREF_1)]. To activate the carboxyl groups, LA was first reacted with similar amounts (2 equiv. / LA) of EDC and NHS for 12 h in dimethyl formamide (DMF). Next, CS-ADH-Rh (0.25 mmol) was added to 10 mL of a 1:1 mixture of DW and DMF, followed by addition of LA-NHS ester (0.35 mmol). After 12 h at 25°C in the dark, the product was purified against DMF (MWCO 3500) and then DW, before being lyophilized. CS-Rh-LA polymers with different ratios of LA to CS-Rh (0.1:1 to 0.3:1) were synthesised, followed by the detection of their critical micelle concentration (CMC).

NMR and FT-IR were used to determine successful conjugation. To record ^1^H-NMR spectra, CS or CS-ADH were dissolved in D2O. To determine ^13^C-NMR spectra, CS-ADH-Rh or CS-ADH-Rh-LA copolymers were dissolved in a mixed solution of DMSO-d6/D2O (1:1, v/v).

**Preparation and characterization** **of non-crosslinked CS-Rh-LA nanoparticles (NC-NPs)**

NC-NPs were constructed using sonication. Briefly, the synthetic CS-ADH-Rh-LA conjugate polymers was dissolved in DW by stirring and sonicated for 12 min using an ultrasonic probe operating at 120 W in 2-s pulses with 4-s intervals. The product was purified by passage through a 0.8 μm membrane filter. To construct cross-linked nanoparticles, a solution of DTT (10% content of ester group in NC-NPs) was added to the NC-NPs after sonication and stirred for 12 h. The NC-NP mixture was dialyzed against DW, filtered and lyophilized. The resulting cross-linked nanoparticles (C-NPs) were stored at 4°C.

To encapsulate DTX in C-NPs, the previously prepared copolymer was mixed with a methanol solution of DTX (100 μL). The solution was added dropwise to 1 mL of CS-Rh-LA in DW and stirred for 5 h at 25°C. The resulting solution was sonicated for 12 min and dialyzed (MWCO 3500) to remove methanol. The cross-linked NPs containing encapsulated DTX (DTX/C-NPs) were prepared following a previously described method [3]. The sample was centrifuged at 4000 rpm for 15 min. The supernatant was recovered and passed through a 0.8 μm membrane filter. All steps were performed in the dark.

The content of DTX was determined by high-performance liquid chromatography (HPLC) using a Hypersil-BDS C18 column (5 μm, 250 mm × 4.6 mm) and a detection wavelength of 227 nm. The mobile phase was composed of a mixture of H_2_O and CH_3_CN (45:55 (v/v)) using a constant flow rate of 1.0 mL/min. DL and EE values were calculated using the following equations:

$$\text{DL\%}=\frac{\text{Weight of DTX in NPs}}{\text{Weight of the inputted conjugates}+\text{weight of DTX in NPs}}\times100\%\text{ (Equation 1)}$$

$$\text{EE\%}=\frac{\text{Weight of DTX in NPs}}{\text{Weight of inputted DTX}}\times100\%\text{ (Equation2)}$$

The *in vivo* stability of C-NPs and NC-NPs was evaluated in PBS and 0.1 M NaCl. The dilution stability against a 1000-fold volume of water was also evaluated. The stability of C-NPs in 10% FBS was also evaluated. The size of the nanoparticles was obtained at 37°C and 100 rpm, and was determined by dynamic light scattering (DLS) at 25°C. The morphologies of C-NPs and NC-NPs were examined by TEM using a model JEM-1200EX II electron microscope (JEOL, Japan).

**Critical micelle concentration (CMC) determination**

CMC was detected by a fluorescence-based method using a pyrene probe [4]. Briefly, pyrene was dissolved in acetone to obtain a final concentration of 2.5×10^–4^ M. Solutions of NC-NPs (1 ng/mL to 1 µg/mL) were added to the pyrene after the acetone was evaporated. A [fluorospectrophotometer](file:///C:/Users/dell/AppData/Local/youdao/dict/Application/8.9.3.0/resultui/html/index.html#/javascript:;) (Hitachi, Tokyo, Japan) was used to record the emission spectra. The results were obtained by plotting I_373_/I_384_ against logarithmic concentrations.

**De-crosslinking and drug release of C-NPs triggered by DTT**

The method has been previously described [5]. Briefly, C-NPs or NC-NPs were dissolved and cultured with 20 μM and 20 mM DTT in [sodium](file:///C:/Users/dell/Desktop/Manuscript%20-2.docx#/javascript:;) [acetate](file:///C:/Users/dell/Desktop/Manuscript%20-2.docx#/javascript:;) solution at 37°C for 24 h. DLS was used to determine the size distributions at different time points. The morphology of the NPs was observed by TEM before or after adding the DTT solution.

The *in vitro* drug release behaviour was investigated in PBS (0.1 M, pH 7.4), PBS containing 20 μM and PBS containing 20 mM DTT. Briefly, the DTX-loaded C-NPs in dialysis bags were added to 35 mL of the particular solution and shaken at 37℃ and 100 rpm for 0.5 h to 72 h. At defined times, 1 mL of the solution was collected and examined by HPLC. The volume of solution was replaced by [isopyknic](file:///C:/Users/dell/AppData/Local/youdao/dict/Application/8.9.3.0/resultui/html/index.html#/javascript:;) blank medium. Each sample was carried out in triplicate.

**Haemolysis assay**

Blood was collected from rabbits, stored in heparinized tubes and centrifuged (3000 rpm, 10 min) to separate red blood cells. The pellet was rinsed with saline three times after the supernatant was discarded using the same centrifugation conditions. The washed red blood cells were resuspended at a final concentration of 2% in saline. A solution of C-NPs (0.1, 0.25, 0.5, 0.75 and 1.0 mg/mL) was added to the obtained erythrocytes and left for 1 h at 37°C. The sample was centrifuged and the supernatant was collected and scanned using a UV-visible spectrophotometer at 541 nm. Each determination was performed in triplicate.

**Intracellular uptake**

To study the intracellular uptake of nanoparticles, the C6 fluorescent model drug was encapsulated into C-NPs (C6/C-NPs). A549 cells were seeded in wells of 12-well plates (1.2×10^4^ cells/well). After growth was 75% confluent, the original medium was discarded and replaced with medium containing 2 μg/mL C6 (free drug group), nanoparticles containing 2 μg/mL C6 (C-NPs group) or medium alone (control group). At different times, the uptake was terminated by thoroughly washing the cells with PBS. Cells were collected in different tubes for flow cytometry (FCM) analysis.

To track the internalisation of C-NPs, confocal laser scanning microscopy (CLSM) was used (Carl Zeiss, Basel, Switzerland) was used. A549 and H1299 cells were incubated in wells of 12-well plates (8×10^3^ cells/well). The fresh media listed above were added until 75% confluence was achieved. Additionally, cells were cultured with free CS (10 mg/mL) 1 h before adding the C-NPs solution (CD44 receptor inhibiting group). After incubation, Hoechst 33342 was used to stain the nuclei. CLSM was performed after washing with PBS three times.

**Transcellular delivery**

The transcellular delivery of C-NPs was investigated in A549 cells. Cells were plated and cultivated on the series of coverslips (I–III; Fig. 4B) at a density of 1×10^4^ cells/well overnight [6]. Cells on coverslip (I) were incubated with C6-loaded C-NPs (2 μg/mL C6) for 4 h. The coverslip was washed with PBS three times, shifted to fresh medium, and cultured with coverslip (II) for 12 h. Identical to the above steps, coverslip (II) was washed and co-cultured with coverslip (III) for a further 12 h. Finally, all the coverslips were rinsed with PBS, stained with Hoechst 33342 for 10 min and observed by CLSM.

***In vitro* cytotoxicity**

Cytotoxicity of Rh, CS-ADH-LA, C-NPs and DTX/C-NPs to A549 and H1299 cells were determined using the CCK-8 kit. Cells were cultivated in wells of a 96-well plate at a density of 5×10^3^ cells/well in 100 μL of complete medium. C-NPs containing 0, 0.1, 0.5, 1, 2, 3, 4, 8, 16, and 32 μg/mL Rh were added and cultivated for 24 h. The cells were washed twice and replaced with fresh medium. To investigate toxicity, the plates were placed in the dark for further 24 h before analysis using the CCK-8 kit. In the SDT group, the cells were exposed to ultrasound (1.2 W/cm^2^ for 1, 3 and 5 min) 24 h prior to the CCK-8 assay. To evaluate synergistic cytotoxicity, cells were incubated with DTX/C-NPs for 24 h (0.001-10 μg/mL Rh, 0.012-120 ng/mL DTX). After rinsing with PBS, the cells were untreated or treated with SDT and cultured as described in the previous step. The results were recorded using a [microplate](file:///C:/Users/dell/Desktop/Manuscript%20-2.docx#/javascript:;) [reader](file:///C:/Users/dell/Desktop/Manuscript%20-2.docx#/javascript:;) at 450 nm. Cell viability was calculated as follows:

The optical density (OD_s_) values represent the absorbance of the samples, while the OD_b_ and OD_c_ signals represent the absorbance of the blank and control groups, respectively.

***In vitro* apoptosis study**

An apoptosis detection kit (Solarbio Life Science, Beijing, China) was used to study apoptosis *in vitro*. A549 cells were exposed to blank medium or medium containing free Rh (2.5 μg/mL), CS-ADH-LA (132 μg/mL), C-NP (2.5 μg/mL of Rh) or DTX/C-NPs (2.5 μg/mL of Rh and 14 μg/mL of DTX) for 12 h. After washing with PBS and replenishing with fresh medium, cells in the SDT groups were continuously ultrasonicated for 3 min. After 4 h, the cells were stained and analysed for apoptosis and necrosis using FCM.

**Detection of** **live/dead cells**

To observe the distribution of live/dead cells, A549 cells were plated on 14 mm-diameter petri dishes (8×10^3^ cells/well) and cultured for nearly 12 h. After treatments with blank medium or medium containing free Rh (2.5 μg/mL), CS-ADH-LA (132 μg/mL) or DTX/C-NPs (2.5 μg/mL of Rh, 14 μg/mL of DTX) for 12 h, the medium was discarded. The cells were washed and then cultured in fresh medium for 4 h. The Calcein-AM/PI Kit was used according to the manufacturer’s instructions. The differential staining of viable and dead cells was recorded using CLSM.

**ROS detection**

Generation and variation of ROS in cancer cells were measured *in vitro* using an ROS detection kit (Beyotime Biotech, Beijing, China). A549 cells were seeded in wells of 12-well plates or in 14 mm-diameter petri dishes at 1.0×10^4^ and 8×10^3^ cells/well, respectively. After 24 h, the medium was replaced with fresh medium alone or medium containing free Rh (2.5 μg/mL) or C-NP (2.5 μg/mL of Rh). After cultivation for 4 h, 10 μM of DCFH-DA diluted with fresh medium was added to the cells. Cells in the SDT groups were treated with ultrasound (1.2 W/cm^2^, 3 min). After a 20 to 30 min incubation, the cells were washed, collected and the fluorescence intensity was determined by FCM. For CLSM observation, the nuclei of treated cells in petri dishes were stained with Hoechst 33342 and observed after washing with PBS. For inhibitory studies, 5 mM NAC as an ROS scavenger was added to the culture medium 1 h before treatment with C-NPs. The samples were observed by CLSM.

Cells were plated on 14 mm-diameter petri dishes (8×10^3^ cells/well) and incubated overnight. First, cells were cultivated with C-NPs at different concentrations of Rh (1, 2.5, and 5 μg/mL) for 12 h. The DCFH-DA probe was loaded as mentioned above. Subsequently, the cells were exposed to ultrasound and sequentially cultured for 4 h. Cell nuclei were stained for CLSM observation. Other cells were incubated with C-NPs containing 5 μg/mL Rh for 2, 4 or 8 h, and treated as described previously.

**Singlet oxygen assay**

The 9,10-dimethylanthracene (DMA) fluorescent probe was used to detect singlet oxygen. Briefly, DMA was dissolved in DMF and added to ethanol along or containing Ce6 (5 μg/mL), Rh (5 μg/mL), C-NPs (containing 5 μg/mL Rh) or EMO (5 μg/mL). The final DMA concentration was 20 μM. After treatment with ultrasound (1.2 W/cm^2^, 3 min) followed by incubation for 6 h, solutions were scanned with a [fluorescence](file:///C:/Users/dell/Desktop/Manuscript%20-2.docx#/javascript:;) [spectrophotometer](file:///C:/Users/dell/Desktop/Manuscript%20-2.docx#/javascript:;) using excitation and emission wavelengths of 360 nm and 380 to 550 nm, respectively. The spectra were recorded to compare the fluorescence intensity.

**Detection of mitochondrial membrane potential (MMP)**

The MMP detection kit that uses a JC-1 probe (Beyotime Biotech, Shanghai, China) was applied. Briefly, cells were plated in wells of 6-well plates (1.2×10/^4^ cells/well) until growth was nearly 75% confluent. The cells were then cultivated in medium alone or medium with DTX/C-NPs containing 2.5 μg/mL Rh and 14 μg/mL DTX for 12 h. After rinsing with PBS, cells were treated with ultrasound (1.2 W/cm^2^) for 1, 3 and 5 min. Twelve hours later, cells were harvested and washed twice, followed by centrifugation. The JC-1 fluorescence probe was added and cells were incubated in the dark for 30 min at 37°C. The cells were washed, harvested and examined by FCM.

**Cell cycle analysis**

The cell cycle was evaluated through FCM using a cell cycle detection kit (Keygen Biotech, Nanjing, China) [7]. First, cells were seeded in 6-well plates (1.2×10/^4^ cells/well) and cultivated overnight. The cells were then incubated with fresh medium alone or medium containing C-NPs (2.5 μg/mL Rh), DTX (14 μg/mL) or DTX/C-NPs (2.5 μg/mL of Rh and 14 μg/mL of DTX) for 12 h. The cells were washed and the medium replaced prior to sonication for 3 min. The cells were cultured for 12 h at 37°C. The cells were harvested and stained with dilute PI/RNase A solution in the dark for 60 min. The results of FCM were analysed using the ModFit LT software to generate the corresponding DNA histograms.

**Cellular microtubule regrowth assay**

The microtubule structure was measured using a Tubulin-Tracker Red (Beyotime Biotech) fluorescence probe and CLSM [8]. Briefly, cells were plated and cultured for 12 h on 14 mm-diameter Petri dishes (8×10^3^ cells/well). The medium was replaced with fresh medium alone or medium containing C-NPs (2.5 μg/mL of Rh), DTX (14 μg/mL) or DTX/C-NPs (2.5 μg/mL of Rh and 14 μg/mL of DTX). The cells were cultured for another 4 h at 37°C. In the following step, cells were rinsed and fixed with paraformaldehyde for 10 min and then stained with the kit. Finally, Hoechst 33342 was used to stain the cell nuclei, and microtubules were acquired.

**Subcellular localisation of nanoparticles**

To observe the subcellular localisation, C-NPs were labelled with C6 by encapsulating them into the hydrophobic core, as mentioned previously. A549 cells were first seeded in petri dishes (8×10^3^ cells/well) for 12 h, then incubated with C6/C-NPs (2 μg/mL of C6) for 1, 3, 6, and 12 h. The cells were rinsed with PBS, fixed and stained with 50 nM Lyso-Track Red (Beyotime Biotech) for 1 h, 20 nM Mito-Tracker Red CMXRos (Beyotime Biotech) for 0.5 h and 166.5 μg/mL Golgi-Tracker Red (Beyotime Biotech) for 0.5 h to monitor the C-NPs distribution in lysosome, mitochondria and Golgi apparatus, respectively. Hoechst 33342 was used to stain nuclei before CLSM.

**Immunofluorescence assay**

A549 cells were seeded on a sterile microscope cover glass (3×10^3^ cells/well) and incubated overnight. The cells were replenished with fresh medium alone or medium containing C-NPs (2.5 μg/mL of Rh) and DTX/C-NPs (2.5 μg/mL of Rh and 14 μg/mL of DTX) for 12 h. Cells were rinsed with PBS. Some SDT groups were treated with ultrasound (1.2 W/cm^2^, 3 min). After 12 h, the cells were fixed and permeabilised using 0.2% Triton X-100. After blocking for 30 min, cells were cultured with GM-130 antibody (DF7556, Affinity Biosciences, Cincinnati, OH, USA) overnight in 1% bovine serum albumin (BSA) dissolved in PBS at 4°C. After washing with 1% BSA, the secondary goat anti-rabbit IgG (H+L) Fluor 594-conjugated-S0006 antibody (Affinity Biosciences) was added. The nuclei of cells were then stained using Hoechst 33342 and examined by CLSM.

**Cell migration and invasion**

Nanoparticles were evaluated for cell migration and invasion as previously described [9]. Briefly, to evaluate cell migration and repair, A549 cells were plated on 12-well plates and cultivated until fully confluent. Sterile 200-μL pipette tips were used to create straight cell-free scratches in the cell monolayers. After being washed and replenished with 2 mL fresh medium or medium including free Rh (0.5 μg/mL), C-NPs (0.5 μg/mL of Rh), DTX/C-NPs (0.01 μg/mL of Rh and 0.056 μg/mL of DTX) or DTX (0.1 μg/mL), the cells were incubated for 24 h. The SDT groups were also treated with ultrasound (1.2 W/cm^2^) for 3 min and incubated for a further 4 h. Images were acquired at 0 h and 28 h after treatment and the wound healing rate was determined.

For the evaluation of invasion, A549 cells were incubated for 12 h with different solutions of fresh medium alone or medium containing C-NPs (0.5 μg/mL of Rh) as well as DTX/C-NPs (0.01 μg/mL of Rh and 0.056 μg/mL of DTX) in Transwell devices. The SDT groups were treated with ultrasound (1.2 W/cm^2^, 3 min). Thereafter, cells were collected and dispersed in FBS-free medium (5×10^4^ cells/well) and placed in the upper chambers. The upper and lower chambers were separated by a membrane whose upper surface was coated with diluted Matrigel (M8370, Solarbio Life Science). Complete culture medium containing 10% FBS in the lower chamber acted as the chemokine. After incubation for 24 h at 37°C, the wells were rinsed before fixing the cells with 4% paraformaldehyde. The upper Matrigel was then wiped off using a [cotton](file:///C:/Users/dell/Desktop/Manuscript%20-2.docx#/javascript:;) [swab](file:///C:/Users/dell/Desktop/Manuscript%20-2.docx#/javascript:;), and crystal violet staining solution (Beyotime Biotech) was applied to stain the cells for 15 min. All images were acquired by inverted microscopy (Nikon Tokyo, Japan).

**Western blot assay to assess SDT induction of apoptosis**

A549 cells were seeded in 10 cm petri dishes (3×10^6^ cells/well) and incubated for 12 h before treatment with free medium or medium containing C-NPs (2.5 μg/mL of Rh) or DTX/C-NPs (2.5 μg/mL of Rh and 14 μg/mL of DTX). The cells were rinsed with PBS and replenished with 10 mL free medium. The SDT groups were treated with ultrasound (1.2 W/cm^2^, 3min). All groups were cultured for 12 h before harvesting and lysis of the cells in RIPA buffer containing 1% phenylmethanesulfonyl fluoride. The variations in apoptosis-related proteins such as MMP9, VEGFA, Caspase-3, and cleaved Caspase-3 were determined by sulphate-polyacrylamide gel electrophoresis followed by transfer of the resolved proteins to polyvinylidene fluoride membranes. The membranes were immunoblotted using the primary antibodies mentioned above, with β-actin as the [internal](file:///C:/Users/dell/Desktop/Manuscript%20-2.docx#/javascript:;) [reference](file:///C:/Users/dell/Desktop/Manuscript%20-2.docx#/javascript:;). Subsequently, horseradish peroxidase-labelled secondary antibodies were co-incubated with the samples to tag the primary antibodies. Finally, protein bands were detected using enhanced chemiluminescence.

**Construction of tumour-bearing mouse models**

To investigate the tumour inhibition effects, bilateral tumour models were constructed for *in vivo* imaging and tumour suppression assays, respectively. First, A549 cells with a density of 1×10^7^ cells/100 μL were injected into the right flanks of mice. The same numbers of A549 cells were injected into the left flanks 4 days after the first injection to construct the bilateral models. After culturing for nearly 3 weeks, the mice were randomly divided into six groups and treated with NS, C-NPs + DTX, Taxotere^®^, DTX + Rh, DTX/C-NPs and DTX/C-NPs + SDT.

***In vivo* biodistribution**

The aim of the bioluminescent imaging system (IVIS Kinetic, Waltham, MA, USA) was to capture the distribution of tumours C-NPs encapsulating the [fluorochrome](file:///C:/Users/dell/Desktop/Manuscript%20-2.docx#/javascript:;) 1,1-dioctadecyl-3,3,3,3-tetramethylindotricarbocyanine iodide (DiR). Briefly, A549 tumour-bearing Barb/c nude mice were subcutaneously injected with A549 cells (1×10^7^/100 μL) into the right armpit. After nearly 3 weeks, mice were intravenously injected with DiR-loaded C-NPs or free DiR, and the DiR fluorescent distribution was observed after 1, 4, 12 and 24 h. The main organs were dissected and imaged after 24 h. In addition, the DiR fluorescence intensity for each group was calculated to determine the accumulation of C-NPs. Data were processed followed by analysis with Living Image 4.1 software.

**Therapeutic efficacy of chemo-SDT**

The preparations were administered to bilateral Barb/c nude mice when the tumours were approximately 150 to 200 mm^3^. Subsequently, all mice were divided into six groups (n = 3 per group): normal saline (NS), C-NPs+SDT, DTX/C-NPs, DTX with Rh, DTX/C-NPs+SDT and Taxotere®. The mice were injected on alternate days starting from day 0 to day 8 with 10 mg/kg DTX. Four hours later, the SDT groups were treated with ultrasound (1.2 W/cm^2^, 3 min). The treatment was directed only at tumours in the right armpit of the mice. Every 2 days, the tumour volumes were recorded and calculated using the formula (width×width×length / 2). Body weights were also determined.

On day 14, all mice were deprived of food but water for 12 h. Blood was collected by extirpating the eyeball on day 15 and serum was isolated. The serum from each mouse was used to test the biochemical profiles (n=3), including alanine aminotransferase (ALT), aspartate amino-transferase (AST), blood urea nitrogen (BUN) and creatinine (CREA). Further, the serum expression of IL-10 and IL-12 was measured using a mouse IL-10 ELISA kit, as well as the mouse IL-12 (p70) ELISA kit (BOSTER Biological Technology Co., Ltd., Santa Clara, CA, USA).

Subsequently, the mice were sacrificed, followed by extraction of the heart, liver, spleen, lung, and kidney tissues. The tissues were embedded in paraffin and sectioned. The sections were stained by hematoxylin and eosin (H&E) staining. To observe tumour apoptosis, tumours in the bilateral mouse model were examined using the TdT-mediated dUTP Nick-End Labelling (TUNEL) assay. The expression of metastasis-associated proteins, including uPA and COX-2, were determined by a western blot assay in both types of tumour tissues. In addition, the tumour tissues were sectioned and stained with anti-CD31 and anti-CD 206 to observe the tumour vessels and expression of [macrophage](file:///C:/Users/dell/Desktop/Manuscript%20-2.docx#/javascript:;)s, respectively.

**Reference**

1. Liu M, Khan AR, Ji J, Lin G, Zhao X, Zhai G. Crosslinked self-assembled nanoparticles for chemo-sonodynamic combination therapy favoring antitumor, antimetastasis management and immune responses. J Control Release. 2018;290:150-164.

2. Liu J, Zhen YZ, Cui J, Hu G, Wei J, Xu R, Tu P, Lin YJ. Dynamic influence of Rhein lysinate on HeLa cells. Int J Oncol. 2018;53:2047-2055.

3. Li L, Liu T, Liao JX, Zhang ZY, Song DB, Wang GH. Dual-responsive TPGS crosslinked nanocarriers to overcome multidrug resistance. J Mater Chem B. 2020;8:8383-8394.

4. Shahzadi I, Asim MH, Dizdarevic A, Wolf JD, Kurpiers M, Matuszczak B, Bernkop-Schnurch A. Arginine-based cationic surfactants: Biodegradable auxiliary agents for the formation of hydrophobic ion pairs with hydrophilic macromolecular drugs. J Colloid Interface Sci. 2019;552:287-294.

5. Huang L, Wang Y, Ling X, Chaurasiya B, Yang C, Du Y, Tu J, Xiong Y, Sun C. Efficient delivery of paclitaxel into ASGPR over-expressed cancer cells using reversibly stabilized multifunctional pullulan nanoparticles. Carbohydr Polym. 2017;159:178-187.

6. Zhou Q, Shao S, Wang J, Xu C, Xiang J, Piao Y, Zhou Z, Yu Q, Tang J, Liu X, Gan Z, Mo R, Gu Z, Shen Y. Enzyme-activatable polymer-drug conjugate augments tumour penetration and treatment efficacy. Nat Nanotechnol. 2019;14:799-809.

7. Alatrash N, Issa FH, Bawazir NS, West SJ, Van Manen-Brush KE, Shelor CP, Dayoub AS, Myers KA, Janetopoulos C, Lewis EA, MacDonnell FM. Disruption of microtubule function in cultured human cells by a cytotoxic ruthenium(ii) polypyridyl complex. Chemical Science. 2020;11:264-275.

8. Brand F, Forster A, Christians A, Bucher M, Thome CM, Raab MS, Westphal M, Pietsch T, von Deimling A, Reifenberger G, Claus P, Hentschel B, Weller M, Weber RG. FOCAD loss impacts microtubule assembly, G2/M progression and patient survival in astrocytic gliomas. Acta Neuropathol. 2020;139:175-192.

9. Li H, Zhang P, Luo J, Hu D, Huang Y, Zhang ZR, Fu Y, Gong T. Chondroitin Sulfate-Linked Prodrug Nanoparticles Target the Golgi Apparatus for Cancer Metastasis Treatment. ACS Nano. 2019;13:9386-9396.
